# Project Brainstorm: Using Neuroscience to Connect College Students with Local Schools

**DOI:** 10.1371/journal.pbio.1001310

**Published:** 2012-04-17

**Authors:** Rafael Romero-Calderón, Elizabeth D. O'Hare, Nanthia A. Suthana, Ashley A. Scott-Van Zeeland, Angela Rizk-Jackson, Aida Attar, Sarah K. Madsen, Cristina A. Ghiani, Christopher J. Evans, Joseph B. Watson

**Affiliations:** 1 Department of Molecular, Cell and Developmental Biology, University of California, Los Angeles, Los Angeles, California, United States of America; 2 Department of Neurology, David Geffen School of Medicine, University of California, Los Angeles, Los Angeles, California, United States of America; 3 Department of Neurosurgery, David Geffen School of Medicine and Semel Institute For Neuroscience and Human Behavior, University of California, Los Angeles, Los Angeles, California, United States of America; 4 Scripps Genomic Medicine, Scripps Translational Science Institute and Scripps Health, La Jolla, California, United States of America; 5 Department of Radiology, Center for the Imaging of Neurodegenerative Disease, University of California, San Francisco, San Francisco, California, United States of America; 6 Interdepartmental Ph.D. Program in Neuroscience, University of California, Los Angeles, Los Angeles, California, United States of America; 7 Department of Psychiatry and Biobehavioral Sciences, University of California, Los Angeles, Los Angeles, California, United States of America; 8 Brain Research Institute, University of California, Los Angeles, Los Angeles, California, United States of America

## Abstract

Neuroscience can be used as a tool to inspire an interest in science in school children as well as to provide teaching experience to college students.

Currently, a large component of college science classes focuses on the acquisition of factual material. Although a solid knowledge base is essential for a successful career in science, the large volume of memorized material tends to make these classes tedious [1]. Importantly, simply knowing facts but not knowing how to apply them or understanding how they are relevant can leave undergraduate students woefully unprepared for the job market or graduate school. A number of initiatives have started to change the focus of science education in college [2],[3], making it more interactive and relevant for students and instructors alike. In fact, this radical shift in teaching science has percolated down the education pipeline to include schools in the kindergarten to 12th grade levels (K-12) [4],[5].

Here we describe a field course called Project Brainstorm that asks third- and fourth-year undergraduate students to apply their knowledge of neuroscience in practice and communicate it effectively to school children in the greater Los Angeles area (see http://www.bri.ucla.edu/bri_education/scienceoutreach.asp). Our model provides undergraduates with a real-world experience in neuroscience and also connects them with the public at large. First, students summarize the structure and function of the brain to their peers, requiring them to fully understand the concepts and most importantly how to effectively speak to school children about how the human brain works in a simple yet accurate way. Second, students must create a hands-on activity that showcases an essential brain function and in turn reflects their solid grasp of the theory behind it. Thus the driving force for the course is that undergraduates test their knowledge of neuroscience as they use it in a real-world context by teaching it (Figure 1).

**Figure 1:**
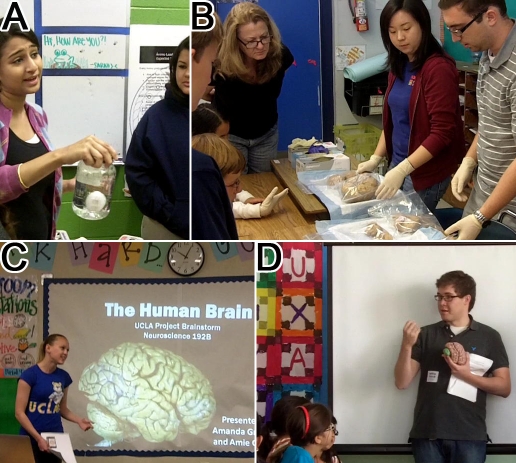
UCLA neuroscience undergraduate students teaching local school children about the brain. (A) Explaining how the water in a jar protects an egg from breaking in much the same way that the cerebrospinal fluid protects the brain from damage. (B) School children look at healthy and diseased human brains wrapped in plastic. (C) Undergraduate neuroscience student introduces the brain to a classroom of seventh grade students. (D) Classroom of fifth grade students learn the gross anatomy of the brain from an undergraduate student holding a model human brain. All participants in this study (legal guardians of school children, undergraduate and graduate students) provided signed consent to publication of their likeness as part of this project.

Project Brainstorm also provides undergraduates with the opportunity to improve their writing and oral communication skills while gaining teaching experience. Perhaps just as important, Project Brainstorm serves as an outreach program that allows undergraduates to interact with both school children and teachers at the K-12 level in the local community, make connections outside the university campus, and explore options for using their degree after graduation. Notably, the course is also designed so that neuroscience graduate students benefit as well from Project Brainstorm. By serving as teaching assistants (TAs), graduate students actively train neuroscience undergraduates and proctor school visits, gaining valuable teaching experience at both the K-12 and college level. Considering the lack of comprehensive teaching opportunities in most graduate programs [6] and the increasing movement of young PhDs into “alternative” careers [7], Project Brainstorm offers a much-needed training opportunity for graduate students.

An essential component of the Project Brainstorm course is the formal guidance that undergraduate students receive in preparing cogent lesson plans. More specifically teams of 2–3 undergraduate students are assigned a K-12 classroom to teach and given a general outline of the material they should master (see course syllabus; Text S1). Subsequently they are expected to create a novel, age-appropriate lesson plan of 45 min to showcase the structure and function of the nervous system. This type of independent, group learning has been shown to increase the retention of facts [8] and encourages students to use their creativity to consolidate their knowledge and identify gaps in their understanding [9]. During the introductory meetings, the TA and course instructor coach students on effective presentation skills such as voice projection, poise, audience engagement, and appropriate use of PowerPoint and/or a black/whiteboard. Similarly, before students are formally allowed to go into the school classrooms, they perform a dress rehearsal of their lesson plan in front of their peer classmates, the TA, the course instructor, and a small panel of invited neuroscience graduate students (including former TAs) and faculty. The practice run allows the presentations to be checked for factual accuracy, appropriateness for the student age group, and improvements in the teaching style. See Text S2 for a sample Microsoft PowerPoint presentation that includes the introduction and brain-in-perspective components.

Assuming that the school children have no significant background in neuroscience, the undergraduate presenter's lesson plan starts with a 5-min introduction of the nervous system. This covers basic gross anatomy (the principal cortical lobes, cerebellum, and brain stem), comparative anatomy, and the structure/function of a neuron. Next, the teams introduce a brain-in-perspective topic to the school children through a 10 min presentation focusing on one age-appropriate topic of interest. Examples include (but are not limited to) senses, memory and learning, motor systems and reflexes, and brain injury (5–9 y of age); any of the previous topics, plus sleep and dreaming, handedness, and pain (10–13 y of age); and any of the previous topics, plus drugs and the brain, nerve impulse conduction, gender differences in the brain, circadian rhythms, stroke, and neurodegenerative diseases (14–18 y of age).

To conclude the lesson plan, the undergraduates guide a 30 min hands-on practicum aided by numerous hands-on teaching props. To encourage active participation in science during the practicum, school students are divided into smaller groups that rotate through a number of different stations, including (a) comparative anatomy (real animal brains, ranging from fruit flies to sheep), (b) human dura matter and spinal cord, (c) human whole brain and sectioned hemispheres, (d) brain injury with pathological human brains sections, and (e) student-developed exercises that highlight their brain-in-perspective topic. See Video S1 to glimpse the hands-on practicum dynamic in the classroom and Text S3 for examples of typical brain-in-perspective activities.

At the conclusion of the course, the undergraduate students submit a 2–3 page instructive lesson plan on their project formatted so that other educators (school teachers, fellow student mentors, or college faculty) can quickly and independently utilize their lesson. These lesson plans (see Text S4 for an example) are simple enough that teachers with limited science backgrounds can implement them on their own within the school's science curricula and can be easily modified to meet the changing needs of the school classroom—two conditions that are thought to improve the efficacy of an outreach program in K-12 schools [10].

We acknowledge that there will be substantial differences in the way the material is delivered in the classrooms, reflecting the styles and personalities of the undergraduates. However, both the quantity and quality of the material are carefully controlled during the practice runs and remain fairly standard between groups. In the process, we have generated an ongoing archive of interactive and concise lesson plans that use the brain as a teaching tool to advocate both the importance and fun of science. Indeed Project Brainstorm's primary goal is not to introduce school children to neuroscience per se but rather to create interaction between college and school students. These interactions will hopefully (1) allow K-12 students to learn about research first-hand and maybe inspire them to pursue higher education and a career in science and (2) provide undergraduates with insight into the educational system and highlight ways in which outreach efforts in general can have a positive impact in society. Moreover, this course format should translate well to any other discipline in the life or physical sciences and could have broad appeal to many educational institutions.

The outreach component of the course has also been quite successful in reaching a large number of school children. In the past five years, over 100 neuroscience undergraduates have completed the Project Brainstorm course and have visited more than 60 classrooms in 30 Los Angeles schools, to reach over 1,900 K-12 students with hands-on and interactive learning experiences focused on neuroscience. This course is unique in its ability to reach large student audiences of varying socioeconomic backgrounds (Table S1). Because a majority of schools in the greater Los Angeles area are classified as Title 1 (at least 40% of its students come from families that qualify as low-income under the United States Census's definitions), we are effectively reaching a large number of students who may not have access to these educational experiences and have fewer opportunities to interact with college students. Although the number of underrepresented minorities earning a science bachelor's degree has risen slightly over the last 15 years, they currently range below 17% of all science degrees conferred in 2007 [11], roughly half of what would be expected based on the demographic representation levels of minorities in the general population [12].

Targeting a younger demographic provides some added advantages. First, school children have an instinctive curiosity about natural phenomena that facilitates their interest and retention of the subject matter [13]. Second, we take advantage of the fact that older students are teaching younger students, a pedagogical strategy that has successfully introduced active science into the classroom in the past [14],[15]. Interestingly, based on anecdotal experiences over the past five years, the younger the student's age (especially 3rd–5th grades), the greater is the student's enthusiasm, spontaneity, questions, and curiosity about neuroscience and the brain. We are currently measuring Project Brainstorm's impact on K-12 students' perception of science, before and after each school visit, in order to provide a more quantitative assessment of our outreach efforts. Additionally, the school presentations will serve as the performance task that helps evaluate the effectiveness of our methodology [16]. Determining how well the K-12 students understood the material during the classroom visits will provide a measure of how successful our program is at both consolidating neuroscience concepts in undergraduate college students and communicating science to school children.

Although the employment prospects for recent college graduates appear to be poor [17], historically evidence shows that having a college degree significantly increases the earning potential and decreases unemployment [18]. Project Brainstorm primarily aims to provide college students practical use of their knowledge through teaching, while reaching out to a newer generation of students to motivate them to pursue a higher education in the sciences. In light of the critical need for properly trained science teachers [19], exposing undergraduate neuroscience students to the K-12 environment can motivate them to consider teaching as a viable and rewarding career. Furthermore, we are also keenly aware that college enrollment in life science majors is dismally low. For instance, in 2003–2004 only 4% of entering college students declared a biological or biomedical science-related major, with only 20% of them successfully completing the bachelor's degree [20]. This lack of interest in science might stem from poor science education and achievement during primary and especially during secondary education [21],[22]. However, this negative influence can be mitigated by allowing young scientists to interact with school children early on [23], something that Project Brainstorm addresses directly.

The importance of science outreach among scientists and science educators has been widely recognized. Indeed, similar programs coupling college faculty and students with schools have been successfully implemented to increase student interest and learning in science [24]. Similarly within the neuroscience community a series of innovative educational initiatives have also been developed over the last decade [25]–[28]. Nevertheless, there remains a persistent resistance to new educational models of instruction [29]. Our hope is that Project Brainstorm offers an additional alternative to make science real for both college undergraduates and school students and bolster the discourse between scientists and the general public.

## Supporting Information

Table S1 **List of schools visited and classrooms taught by Project Brainstorm during the 2006–2011 school years within the Greater Los Angeles Area.** Elementary Schools represent kindergarten through fifth grade (5–10 y of age); Middle schools represent sixth through eighth grades (11–13 y); High schools represent ninth through 12th grades (14–18 y); multi-level schools represent kindergarten through eighth grade (5–13 y). *Title I school (at least 40% of students come from families that qualify as low-income under the United States Census definitions). ^†^School visited multiple times. ^#^ 2009–10 school year data presented (except for Hawthorne Math & Science Academy and Animo Leadership Charter, for which 2008–2009 data were used), and it is representative of the 2006–2011 time period when schools were visited. The main five ethnic/racial groups are shown. AI, American Indian/Alaskan. The heading Asian includes Filipino and Pacific Islanders. The heading White only includes non-Hispanic White students. N/A, data not available or missing. Not all percentage totals will equal 100 since other ethnicities are not shown. Total number of students and ethnicity profiles were obtained by referring to the School Accountability Report Cards (SARC), which can be viewed at http://notebook.lausd.net/schoolsearch/selector.jsp (for the Los Angeles Unified School District), at http://www.smmusd.org/ (for the Santa Monica-Malibu Unified School District), at http://ccusd.org/ (for the Culver City Unified School District), at http://www.hawthorne.k12.ca.us/ (for the Hawthorne School District), and at http://www.icefla.org/ (for the ICEF Public Schools).
(RTF)

Text S1 **Course syllabus for Project Brainstorm.**
(RTF)

Text S2 **Representative PowerPoint presentation of a Project Brainstorm school visit.**
(PPT)

Text S3 **Example brain-in-perspective topics for three different age groups.**
(RTF)

Text S4 **Example of a complete lesson plan for use in schools by teachers.**
(RTF)

Video S1 **Project Brainstorm classroom visit highlights.**
(WMV)
