# Supplementary material for: Project Brainstorm: Using Neuroscience to Connect College Students with Local Schools
Source: PLoS Biol. 2012 Apr 17;10(4):e1001310. doi: 10.1371/journal.pbio.1001310 (PMC3328426; doi:10.1371/journal.pbio.1001310)
Supplement: Table S1 — List of schools visited and classrooms taught by Project Brainstorm during the 2006–2011 school years within the Greater Los Angeles Area. Elementary Schools represent kindergarten through fifth grade (5–10 y of age); Middle schools represent sixth through eighth grades (11–13 y); High schools represent ninth through 12th grades (14–18 y); multi-level schools represent kindergarten through eighth grade (5–13 y). *Title I school (at least 40% of students come from families that qualify as low-income under the United States Census definitions). †School visited multiple times. # 2009–10 school year data presented (except for Hawthorne Math & Science Academy and Animo Leadership Charter, for which 2008–2009 data were used), and it is representative of the 2006–2011 time period when schools were visited. The main five ethnic/racial groups are shown. AI, American Indian/Alaskan. The heading Asian includes Filipino and Pacific Islanders. The heading White only includes non-Hispanic White students. N/A, data not available or missing. Not all percentage totals will equal 100 since other ethnicities are not shown. Total number of students and ethnicity profiles were obtained by referring to the School Accountability Report Cards (SARC), which can be viewed at http://notebook.lausd.net/schoolsearch/selector.jsp (for the Los Angeles Unified School District), at http://www.smmusd.org/ (for the Santa Monica-Malibu Unified School District), at http://ccusd.org/ (for the Culver City Unified School District), at http://www.hawthorne.k12.ca.us/ (for the Hawthorne School District), and at http://www.icefla.org/ (for the ICEF Public Schools). (RTF) [file pbio.1001310.s001.rtf]

Table S1.

	School Name	Grade(s) taught	Number of students taught	Total Number of students in school#	Racial/Ethnic Profile (%)#	
					AI	Asian	Black	Hispanic	White	
Elementary Schools	59th St.*	1, 2	60	401	0.7	1.0	45.1	52.9	0.2	
	Brentwood Science Magnet*†	5	61	1279	0.7	21.1	17.4	46.9	13.8	
	El Marino Language	5	120	734	0.14	23.6	7.0	29	36	
	Fairburn†	5	102	419	1.0	23.6	1.9	5.7	67.8	
	Franklin Ave.*†	3, 5	90	437	0.5	15.5	1.8	30.7	51.5	
	McKinley*	5	70	889	0.0	0.0	16.9	83.1	0.0	
	Nestle*†	2, 3, 5	120	518	0.2	4.3	3.7	10.4	81.5	
	Nora Sterry*	4, 5	86	339	1.2	3.8	10.3	74.6	10.0	
	Overland	5	50	470	0.6	19.6	8.7	12.3	58.7	
	Roosevelt†	5	114	789	0.5	10.3	4.1	11.8	64.8	
	UCLA Lab	3	25	N/A	N/A	14.0	10.0	32.0	36.0	
	Webster	3	60	400	0.5	5.5	1.3	8.0	81.5	
Middle Schools	Culver City*†	6-8	99	1567	0.0	14.4	20.0	42.0	24.0	
	Emerson*†	7	40	992	0.9	8.3	21.5	52.5	16.8	
	Le Conte*	6-8	90	1428	0.4	7.0	2.2	79.1	11.3	
	Paul Revere Math/Science Magnet	7	30	431	0.7	11.2	12.3	36.7	39.2	
	South Gate*†	7	60	2805	0.2	0.1	0.2	99.2	0.3	
	View Park Prep Accelerated Charter*†	7	96	333	0.6	0.6	97.9	0.9	0.0	
High Schools	Animo Leadership Charter*	12	40	574	0.2	0.2	0.5	98.8	N/A	
	Fremont*	10-12	30	4540	0.2	0.1	8.9	90.6	0.2	
	Dorsey*	9	15	1698	0.2	0.5	56.5	42.6	0.2	
	Grant*†	9-11	90	2653	0.3	4.1	4.8	63.2	27.6	
	Hamilton*	12	60	3107	0.5	5.3	28.5	48.7	17.2	
	Hawthorne Math & Science Acad.*	11, 12	34	581	0.3	10.3	11.7	72.5	2.4	
	Helen Bernstein*	10-12	40	1482	0.1	6.8	2.6	81.9	8.4	
	Lou Dantzler Preparatory*	10	20	267	0.0	1.5	95.4	0.4	0.0	
	Santa Monica	9, 10	60	3106	0.0	7.0	8.0	38.0	40.0	
	University*†	10-12	90	2253	0.1	12.5	16.3	62.5	8.6	
Multi-Level Schools	St. Cyril of Jerusalem	5	29	N/A	N/A	N/A	N/A	N/A	N/A	
	Turning Point	5	32	N/A	N/A	N/A	N/A	N/A	N/A	
										
