# Supplementary material for: Project Brainstorm: Using Neuroscience to Connect College Students with Local Schools
Source: PLoS Biol. 2012 Apr 17;10(4):e1001310. doi: 10.1371/journal.pbio.1001310 (PMC3328426; doi:10.1371/journal.pbio.1001310)
Supplement: Text S1 — Course syllabus for Project Brainstorm. (RTF) [file pbio.1001310.s002.rtf]

Text S1.

Course syllabus for Project Brainstorm.


Neuroscience 192B Neuroscience Outreach
Project Brainstorm 
Spring 2011 Syllabus

Course Instructors: 	Joseph Watson, Ph.D. and Cristina Ghiani, Ph.D.
TA / Coordinator: 	Sarah Madsen (Office: NRB 225E)
Classroom: 		NRB 368
Contact information: brainstorm@ucla.edu
	    
COURSE DESCRIPTION
Project Brainstorm is the K-12 science education outreach program of the Brain Research Institute (BRI) and the Neuroscience Interdepartmental Ph.D. and Undergraduate Programs.  The goal of Project Brainstorm is to stimulate interest in science for children and adolescents in grades K-12 by providing hands-on learning experiences that emphasize the function and importance of the brain. 

Project Brainstorm is coordinated by graduate students in the Neuroscience Interdepartmental Ph.D. Program, and carried out by teams of undergraduate students who visit Los Angeles area schools throughout the year.  A typical visit involves group participation, interactive games, and hands-on exercises with teaching props, brain models and real animal and human brains.

The first half of the quarter is devoted to developing and practicing your own brain lessons that you will present at local K-12 schools during the last five weeks of the quarter.  In small groups (two or three students), you will select a brain topic that you think is exciting and important and we will guide you in preparing your 45-minute presentation, which will be aimed at either a elementary, middle, or high school class. These lesson plans will include a general introduction to the nervous system, leading to a more specific example of the importance of the brain in everyday life accompanied by a fun and educational hands-on activity. Past topics have included diseases (addiction, Alzheimer's, etc.), learning and memory, spinal cord injury, development and aging, and sleep, for example.

COURSE GOALS
Students will:
·	Connect their academics and the “real world” using their community experiences.
·	Gain teaching experience at the K-12 level.
·	Improve writing and communicating skills.
·	Explore options for using a science degree after graduation.

COURSE POLICIES
Both your TA and your faculty sponsor will evaluate you for your grade. Grading is Pass/No Pass for 4 units, which will count as general units. You must complete all course assignments and requirements in order to receive a passing grade (attend and participate in class meetings, complete journals and final writing assignment, and participate in ALL school visits). Units will not count toward satisfaction of the specific course requirements of the Neuroscience major in the Interdepartmental Undergraduate Program for Neuroscience. The close relationship between the student and the coordinator allows for strong evaluations of strengths and weaknesses. Thus, at the end of the quarter all students can ask for written evaluations of their performances that will then be kept on file in the office.

ASSIGNMENTS AND REQUIREMENTS
Lesson Plan: Students will be paired into groups and assigned to either an elementary, middle, or high school class. Based on the age group of their class, they will prepare a relevant 45min lesson plan that will showcase the structure and function of the nervous system. The lesson plan will consist of three parts: 
1)	An introduction to the nervous system (5 minutes). You should review general anatomy (the lobes of the brain, brain stem, and spinal cord along with their major functions) and the structure and function of neurons, so that your students will have a basic understanding of the brain and are prepared for the main topic of your lesson.
2) A presentation that places the brain in perspective (10 minutes).  Why is it important or amazing? How does your brain take care of all the things you do? How can you damage or protect it? etc...  Your Choice!
Here are some ideas of topics that are appropriate for the following age groups but feel free and encouraged to create your own ideas:


Elementary school (grades 1-5; 5-9 years):
 senses, 		            
 memory and learning             
 motor systems and reflexes  (spinal cord)                                    
  brain injury

Middle school (grades 6-8; 10-13 years): 
all of the previous, plus:                       
 sleep and dreaming                 
 exercise                                   
 creativity

High school  (grades 9-12; 14-18 years): 
all of the previous, plus:                     
 drugs and the brain                  
 stress and the brain                  
 circadian rhythm
*Presentation topics cannot be shared by more than one group- each group must have a different topic.
3) A hands-on practicum that will include teaching props, brain models and real animal and human brains.   There will be a total of 6 (10-minute) stations as follows (30 minutes total):
	a) Brain evolution, animal brains
	b) The human brain, whole & hemispheres, dura matter, spinal cord
	c) Neural transduction, neuron structure and AP
	d) Brain damage: egg/jar + slices
e)  Learning: prism goggles
f) Free. Undergraduates will develop a station that is relevant to their brain-in-perspective topic.
The lesson plans must be completed by the end of the 4th week.

Writing Assignments: Students are required to write weekly journals (1 page) outlining the progress of their lesson plans and/or the teaching experience. They are due Monday at 5pm by email (brainstorm@ucla.edu).  No late journals will be accepted.

Students are also required to write a final written assignment, which will be due at the end of the quarter. The final assignment should be a 2-3 page instructive lesson plan on their project formatted so that others (teachers, students, faculty) would be able to implement the lesson they developed over the course.

Group Meetings: During the first half of the quarter, class will meet to discuss and present lesson plans. These sessions will run between 1-2 hours. Additional one-on-one appointments with the TA (15-20 min) will be scheduled by each group before they present to finalize your lesson plans or discuss further developments in your presentation.

Classroom Teaching: In the last half of the quarter, each group will present their lesson plan at their assigned school. Every student is expected to attend all teaching sessions to help out with the hands-on practicum (there will be ~5 teaching sessions). During the 9am-12pm Wed class time, we will meet on campus, drive to a school, setup, present, cleanup, and return to campus. Please be advised that you require some flexibility during these weeks, to visit the schools during the days and times that they allow us.


QUARTER SCHEDULE

Week	Date (Wed)	Assignment	Due	
1	March 30	Class Meeting 1:  Introduction to the course. Group formation and assignment of grades to teach. Details on how to prepare the lesson plan (format, length, style). Resources to use to prepare the lesson plan.
Group Journal #1: Outline the introduction and brain in perspective (BIP) parts of your lesson plan. What will be your BIP topic and what will your take home message for the topic? Is the information relevant to the age group of your students? What information is age-sensitive? Is this information fun and interesting? Is it going to engage the students' interest in the brain? How are you presenting the information?  What sources will you use for your info (include 3).	Course Contract
Liability Waiver	
2	April 6
	Class Meeting 2: Review of your proposed lesson plan. You should have a complete draft of your lesson plan by now. The course coordinators will go over the plan and advise on changes that should be made.
Group Journal #2: Outline the 3rd part of your lesson plan (the hands on activity). What exactly will you do and say for the 5th station? How does this relate to your presentation?  What props will you need?
Since you will rotate presenting each of the permanent stations, outline the information you will describe at each station. Give entertaining facts!	Monday: 
Group Journal #1
Wednesday: 
Course Contract
Liability Waiver	
3	April 13	Class Meeting 3: Presentation of your revised lesson plans to your peers and course coordinators only. After the presentations, briefly describe the hands on activity, information provided and the take home message to your peers. 
Group Journal #3: Come up with and describe a shorter activity (~3-4min, like the egg demo) and a longer activity (~7-8mib).  Include materials needed. 	Monday: Group Journal #2	
4	April 20	Class Meeting 4: Dress Rehearsal. Presentation of your lesson plans to your peers, the course coordinators and a selected panel of faculty. The faculty will be there to offer helpful suggestions	Monday: 
Group Journal #3	
5	April 27	1st School Visit: Group 1 will present their lesson plan to the school grade assigned. All other groups will help out during the 'hands-on' practicum.
Presenter Individual Journal: Discuss the teaching session. How effective do you think it was? What went right/wrong? How could you improve your lesson plan? Did you get your take home message across?
Helper Individual Journal #1: What seemed to engage the students the most? Why do you think it was most engaging? What engaged the least? How can this be modified to make it exciting, give specific examples and modifications.
		
6	May 4	2nd School Visit: Group 2 will present their lesson plan to the school grade assigned. All other groups will help out during the 'hands-on' practicum.
Helper Individual Journal #2: Find a resource/website/podcast… that could be beneficial to the next NS192B class and come up with a short activity or topic/presentation that it could be used for.
	Monday: 
Presenter Individual Journal OR Helper Individual Journal #1
	
7	May 11	3rd School Visit: Group 3 will present their lesson plan to the school grade assigned. All other groups will help out during the 'hands-on' practicum.
Helper Individual Journal #3: Discuss why you decided to major in science in general and neuroscience in specific.  Relate your experiences to what could be done for younger kids to get them excited about science and the brain.	Monday: 
Presenter OR Helper Individual Journal	
8	May 18	4th School Visit: Group 4 will present their lesson plan to the school grade assigned. All other groups will help out during the 'hands-on' practicum.
Helper Individual Journal #4: Discuss what you learned from this class about teaching and about yourself as a teacher. How can the class be improved? Strengths and weaknesses of class, be honest? How can we reach and recruit more undergrads?	Monday: 
Presenter OR Helper Individual Journal	
9	May 25	5th School Visit: Group 5 will present their lesson plan to the school grade assigned. All other groups will help out during the 'hands-on' practicum.
	Monday: 
Presenter OR Helper Individual Journal 	
10	June 1	6th School Visit: Group 5 will present their lesson plan to the school grade assigned. All other groups will help out during the 'hands-on' practicum.
	Monday: 
Presenter OR Helper Individual Journal Friday: Final Assignment	
Class Schedule:

Week 1:		9:30am-12pm	NRB 368

Weeks 2-3: 		10am-12pm 	NRB 368

Week  4:     		9:30am-12pm	NRB 368 (Dress rehearsal) 

Classroom Visits:

Week 5 (April 27th):	9am-12:30pm	View Park Prep Middle School (7th grade, 28 studetns)

Week 6 (May 4th):	9am-12:30pm	Dorsey High School (9th grade, 15 students)

Week 7 (May 11th):	9am-12:30pm	Animo Locke High School (12th grade, 30-50 students)

Week 8 (May 18th):	9am-12:30pm	 Culver City Middle School (7th grade, 34 students)

Week 9 (May 25th):	 9am-12:30pm	El Marino Language School (5th grade, 120/auditorium or 
56/classroom)

Week 10 (June 1st):	9am-12pm	McKinley Elementary School (5th grade, 70 students)

Finals Week:		no class meeting

 
