# Supplementary material for: Project Brainstorm: Using Neuroscience to Connect College Students with Local Schools
Source: PLoS Biol. 2012 Apr 17;10(4):e1001310. doi: 10.1371/journal.pbio.1001310 (PMC3328426; doi:10.1371/journal.pbio.1001310)
Supplement: Text S2 — Representative PowerPoint presentation of a Project Brainstorm school visit. (PPT) [file pbio.1001310.s003.ppt]

## Slide 1
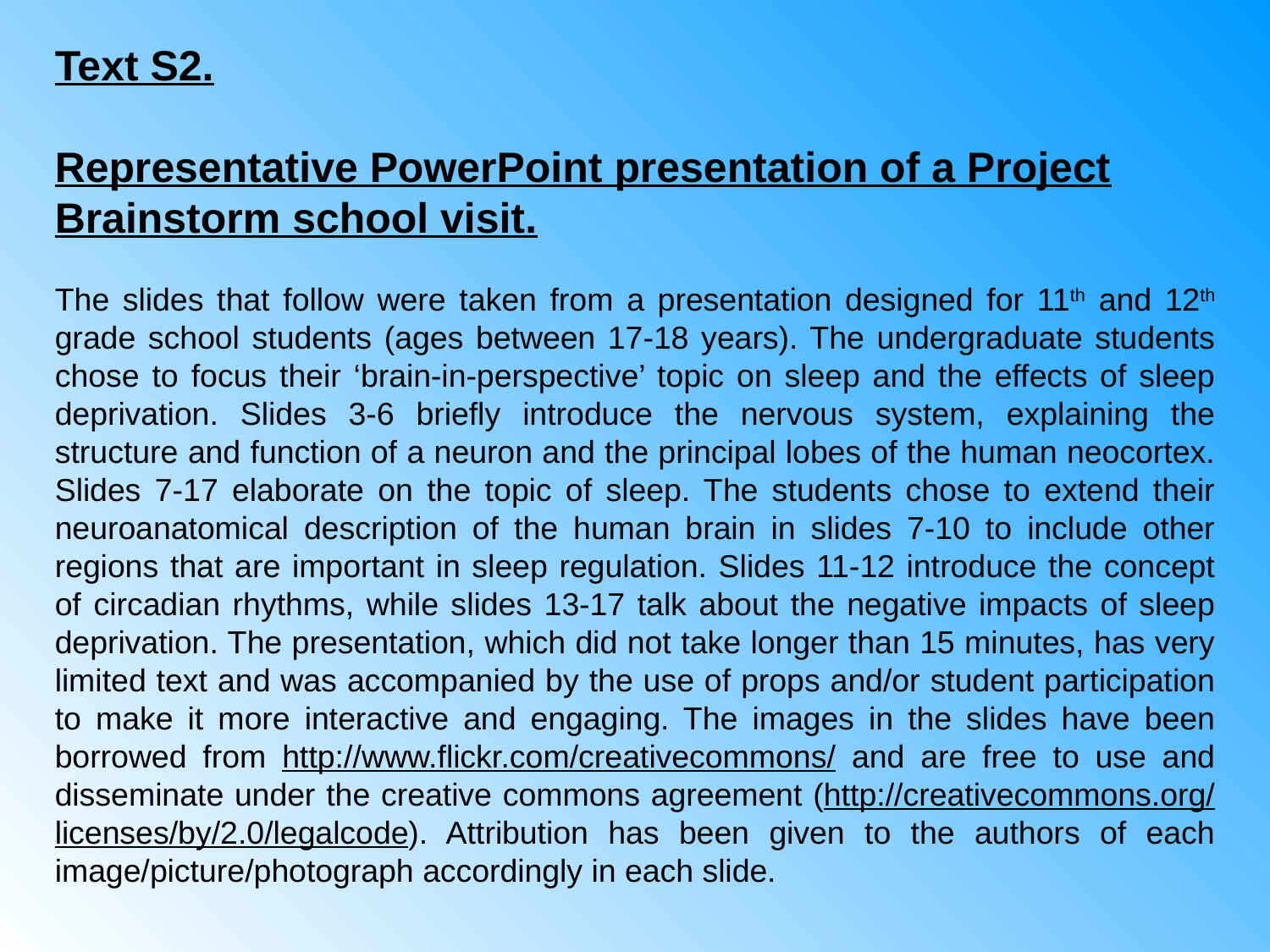

Text S2.
Representative PowerPoint presentation of a Project Brainstorm school visit.
The slides that follow were taken from a presentation designed for 11th and 12th grade school students (ages between 17-18 years). The undergraduate students chose to focus their ‘brain-in-perspective’ topic on sleep and the effects of sleep deprivation. Slides 3-6 briefly introduce the nervous system, explaining the structure and function of a neuron and the principal lobes of the human neocortex. Slides 7-17 elaborate on the topic of sleep. The students chose to extend their neuroanatomical description of the human brain in slides 7-10 to include other regions that are important in sleep regulation. Slides 11-12 introduce the concept of circadian rhythms, while slides 13-17 talk about the negative impacts of sleep deprivation. The presentation, which did not take longer than 15 minutes, has very limited text and was accompanied by the use of props and/or student participation to make it more interactive and engaging. The images in the slides have been borrowed from http://www.flickr.com/creativecommons/ and are free to use and disseminate under the creative commons agreement (http://creativecommons.org/ licenses/by/2.0/legalcode). Attribution has been given to the authors of each image/picture/photograph accordingly in each slide.

## Slide 2
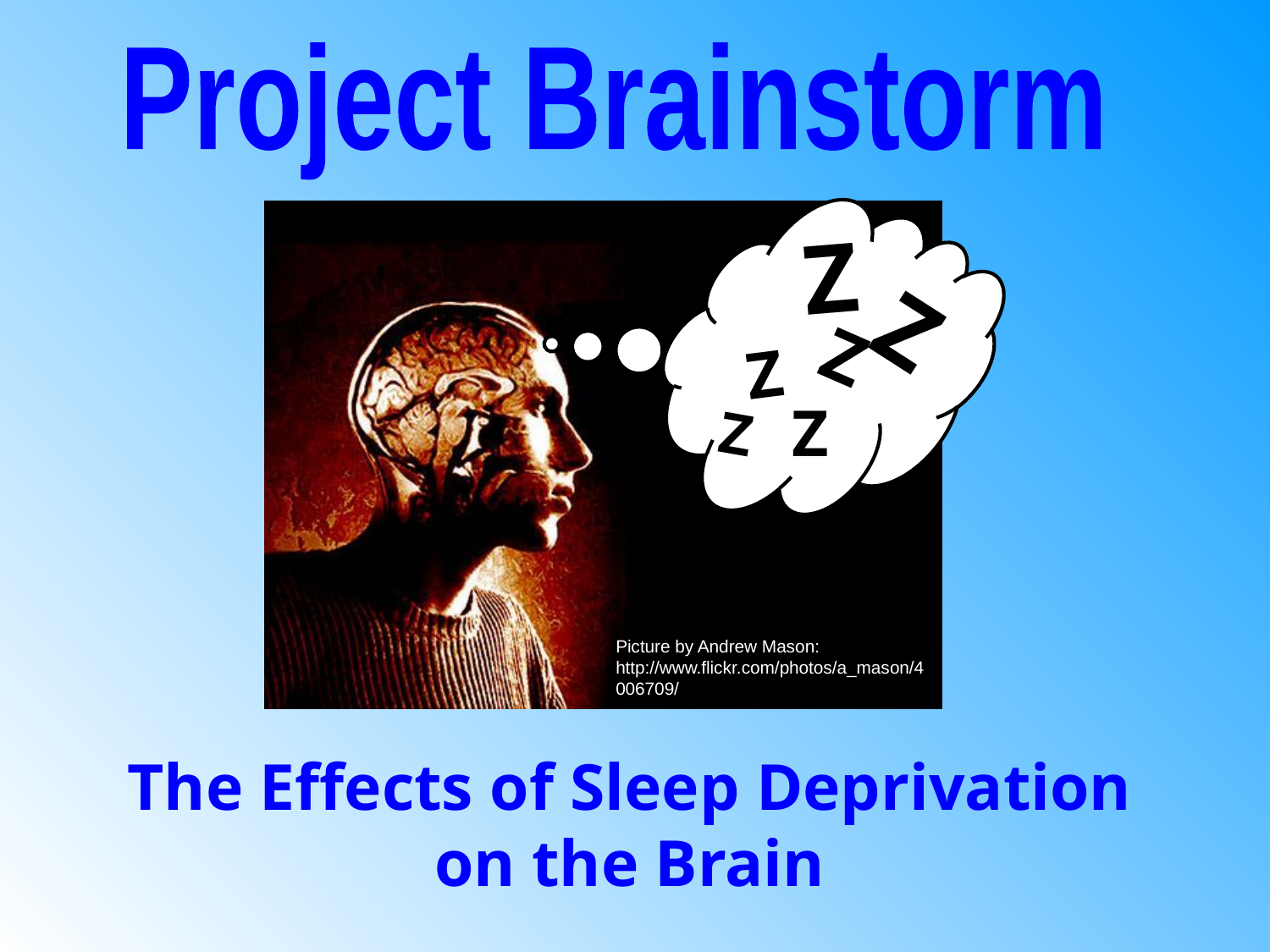

Project Brainstorm
Z
Z
Z
Z
Z
Z
Picture by Andrew Mason: http://www.flickr.com/photos/a_mason/4006709/
# The Effects of Sleep Deprivation on the Brain

## Slide 3
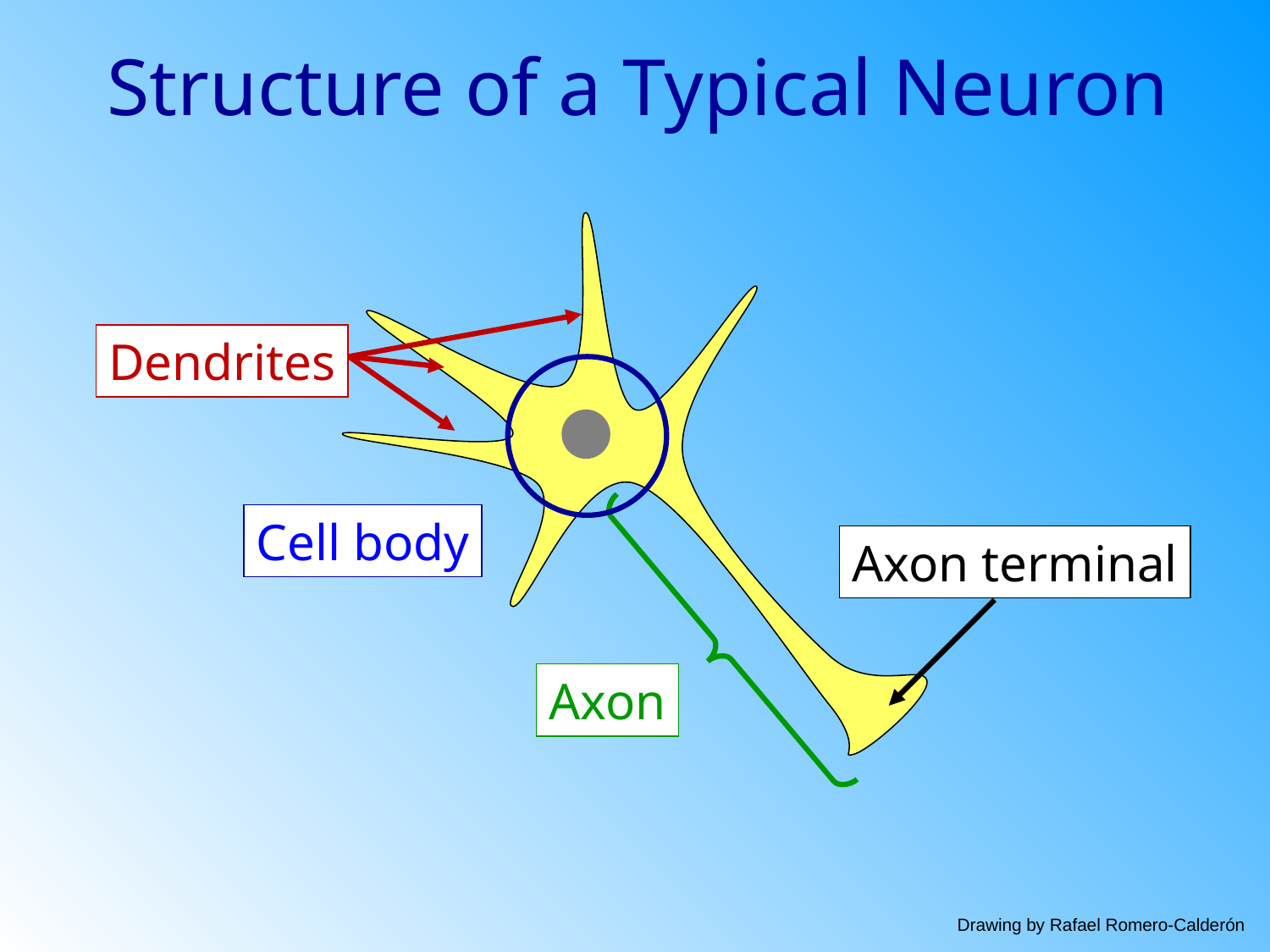

Structure of a Typical Neuron
Dendrites
Cell body
Axon terminal
Axon
Drawing by Rafael Romero-Calderón

## Slide 4
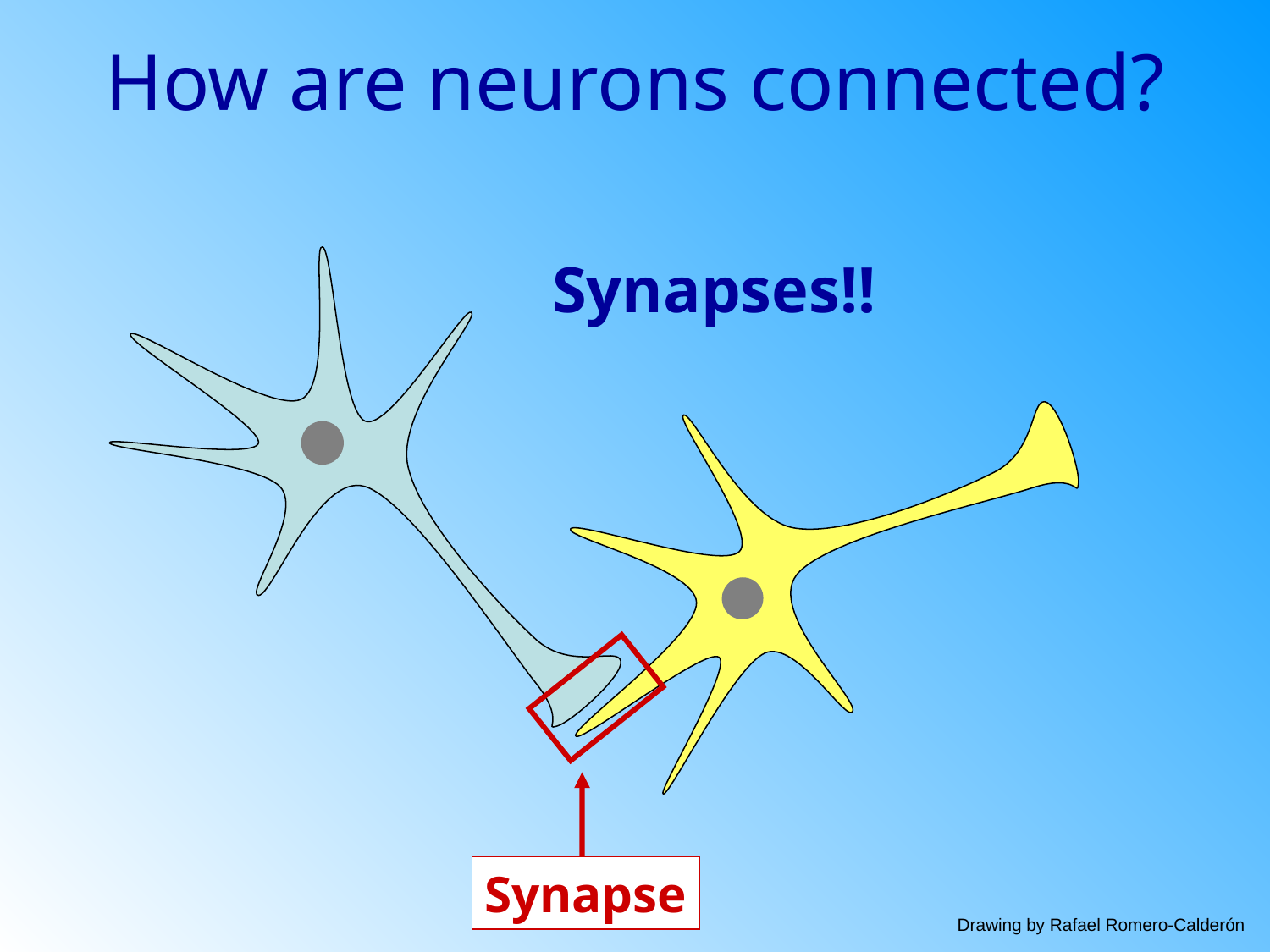

# How are neurons connected?
Synapses!!
Synapse
Drawing by Rafael Romero-Calderón

## Slide 5
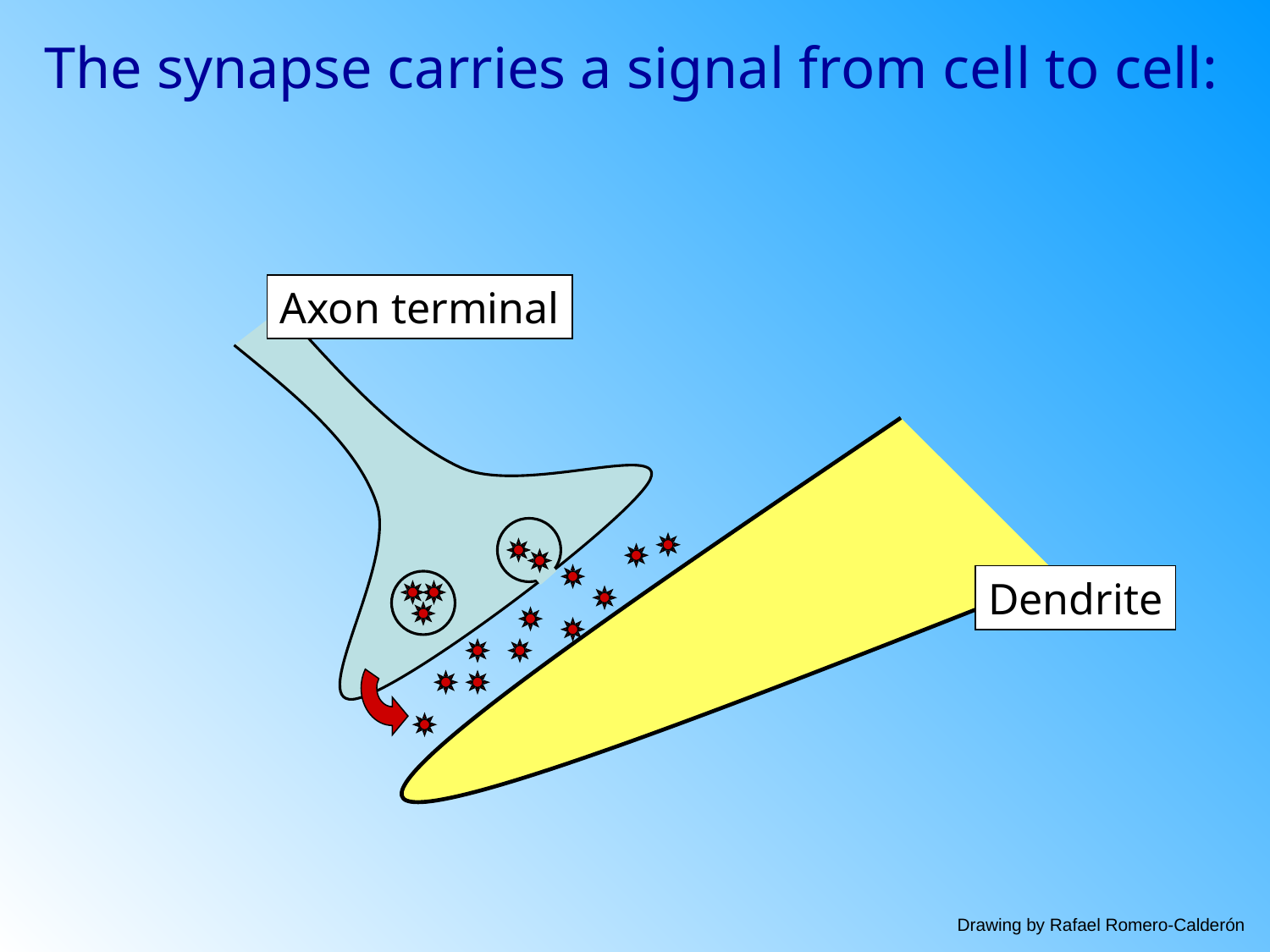

The synapse carries a signal from cell to cell:
Axon terminal
Dendrite
Drawing by Rafael Romero-Calderón

## Slide 6
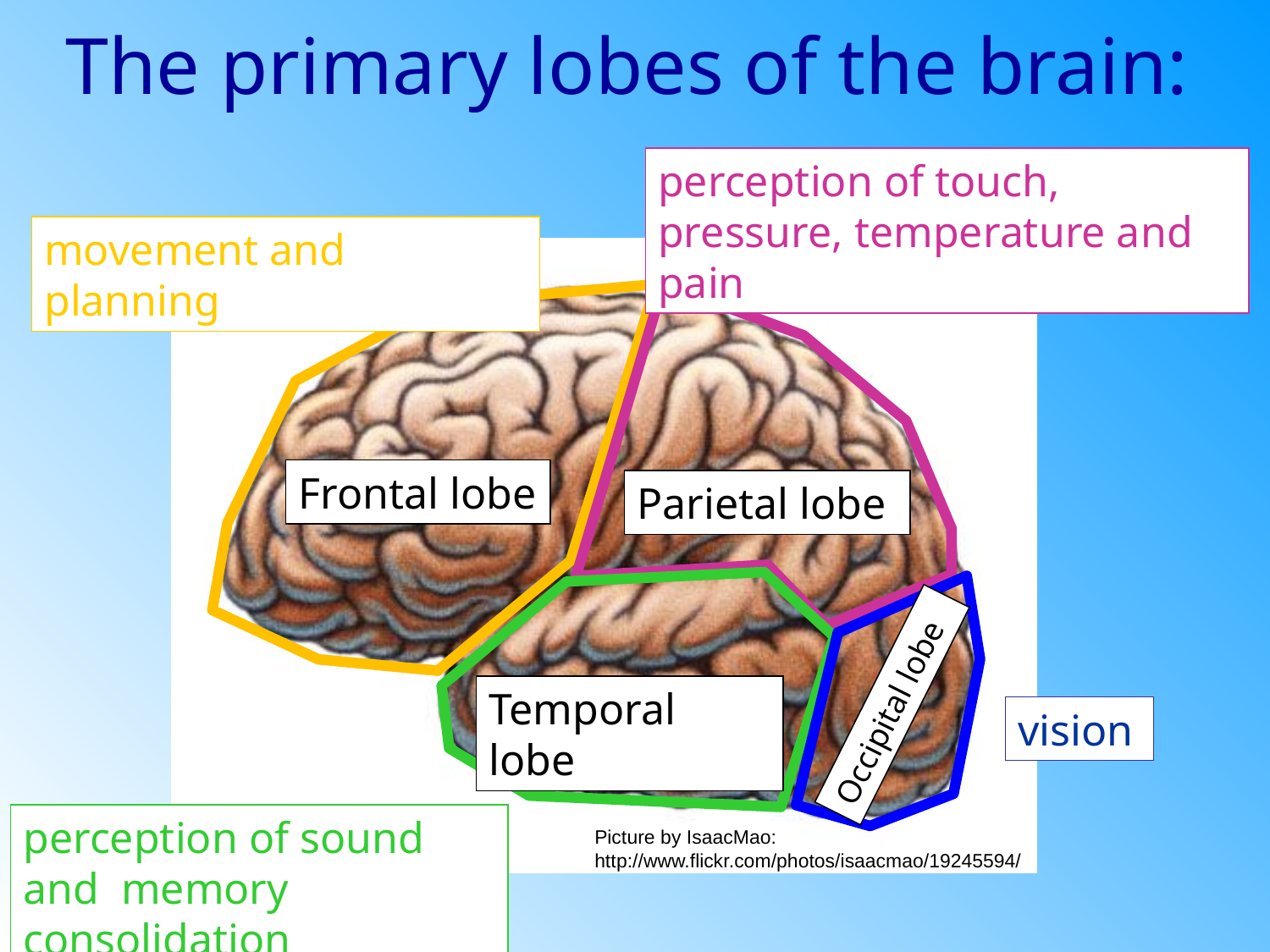

The primary lobes of the brain:
perception of touch, pressure, temperature and pain
movement and planning
Frontal lobe
Parietal lobe
Temporal lobe
Occipital lobe
vision
perception of sound and memory consolidation
Picture by IsaacMao: http://www.flickr.com/photos/isaacmao/19245594/

## Slide 7
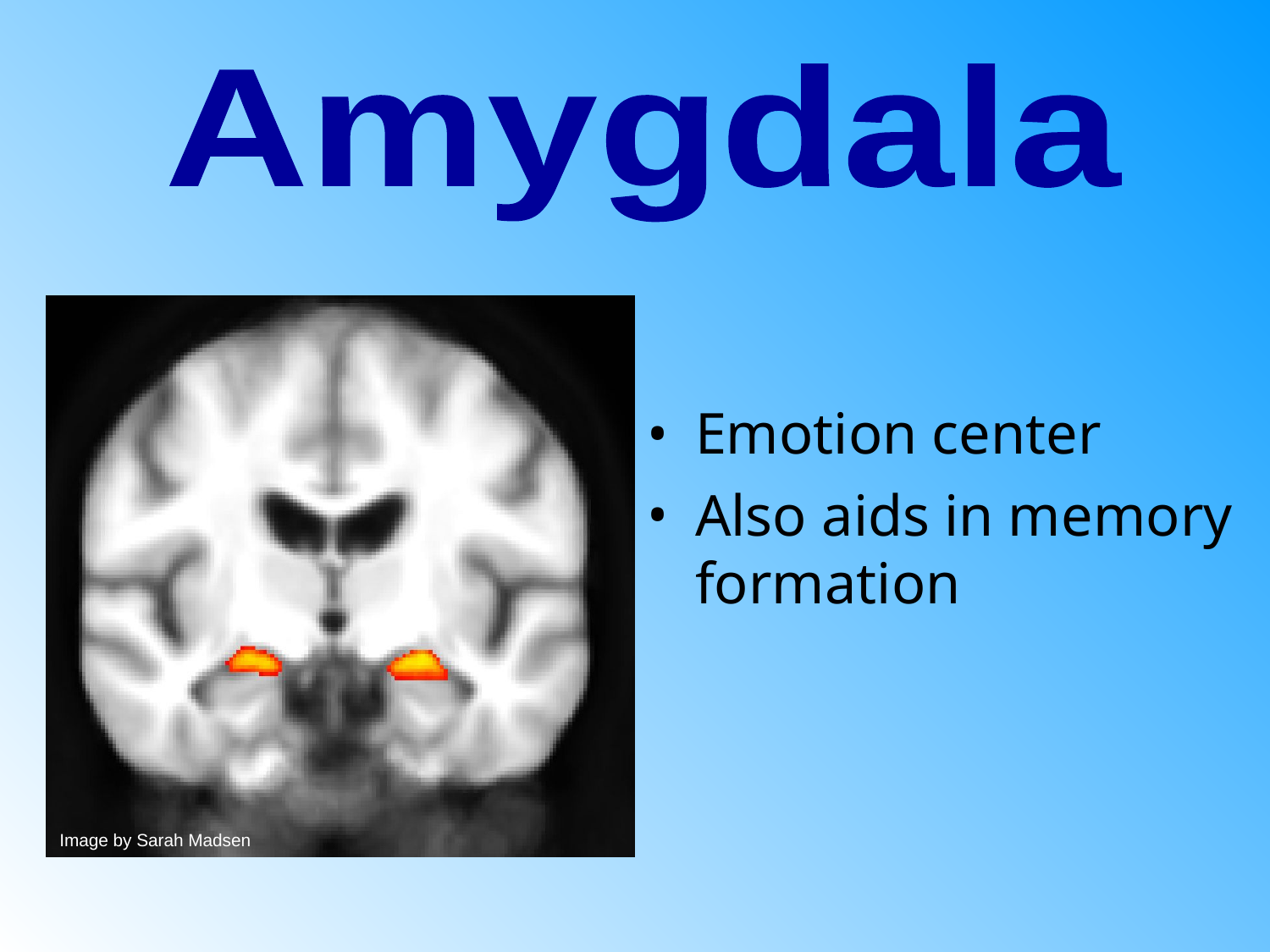

Amygdala
# Emotion center
Also aids in memory formation
Image by Sarah Madsen

## Slide 8
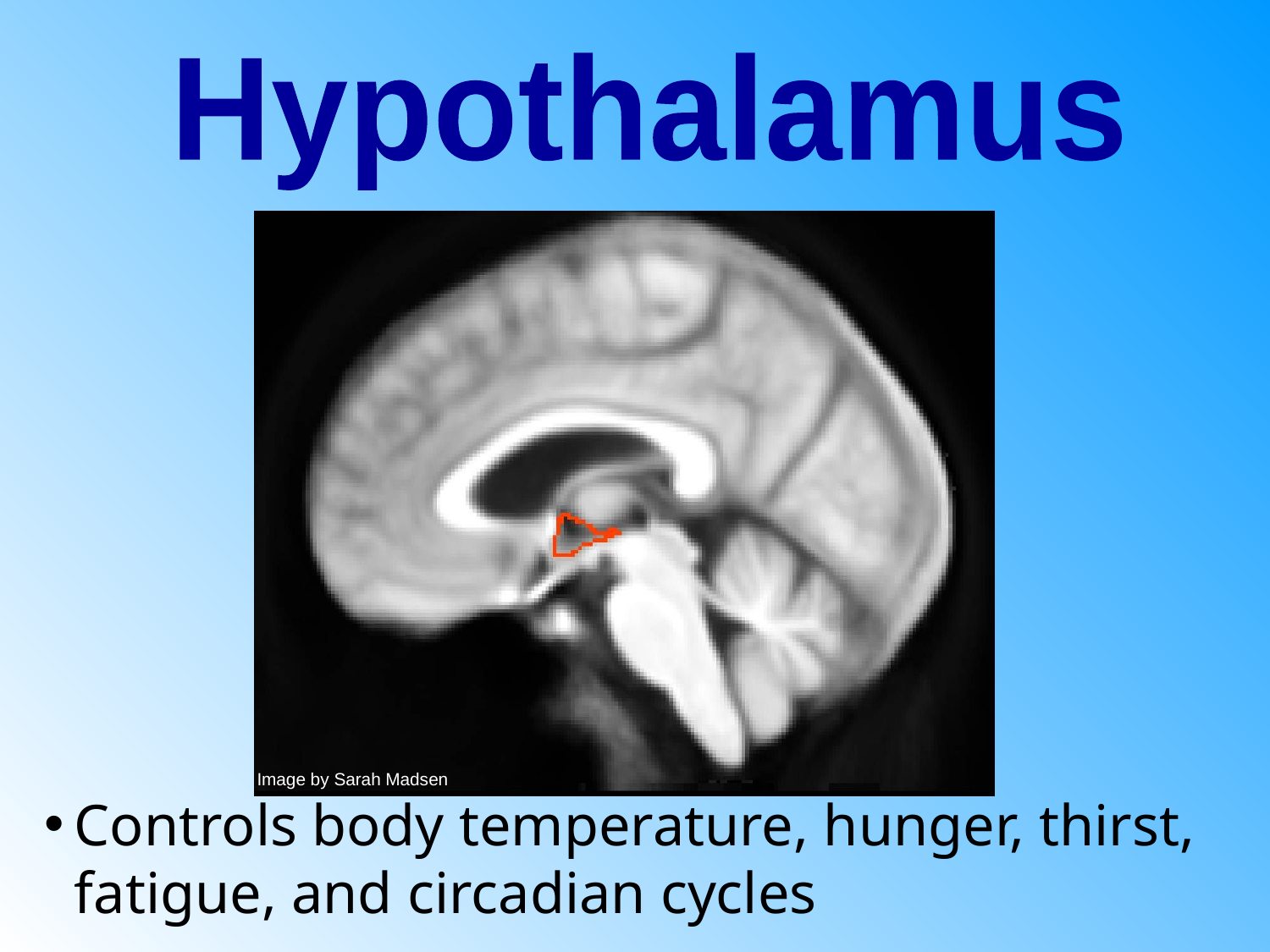

Hypothalamus
Image by Sarah Madsen
Controls body temperature, hunger, thirst, fatigue, and circadian cycles

## Slide 9
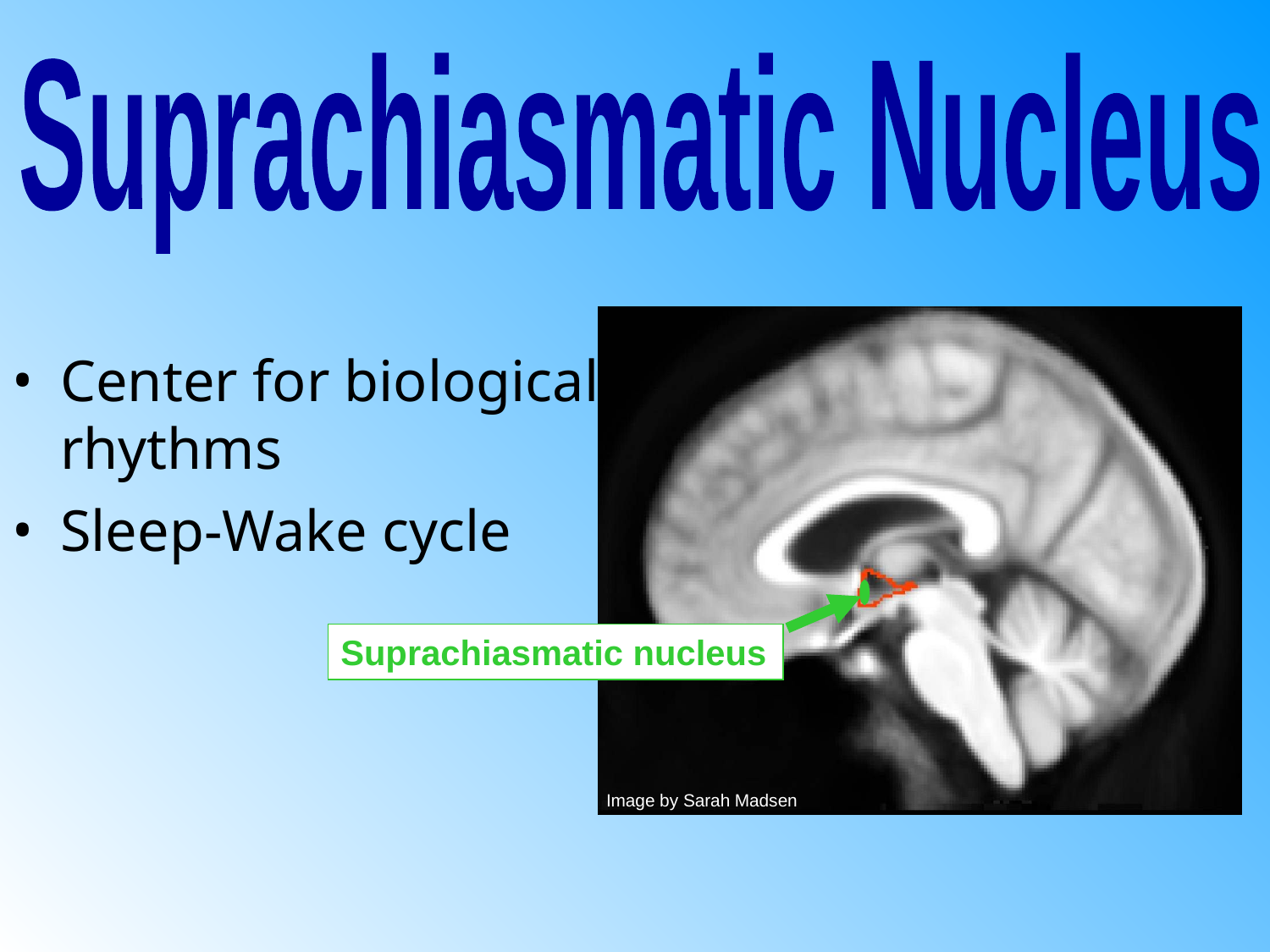

Suprachiasmatic Nucleus
# Center for biological rhythms
Sleep-Wake cycle
Suprachiasmatic nucleus
Image by Sarah Madsen

## Slide 10
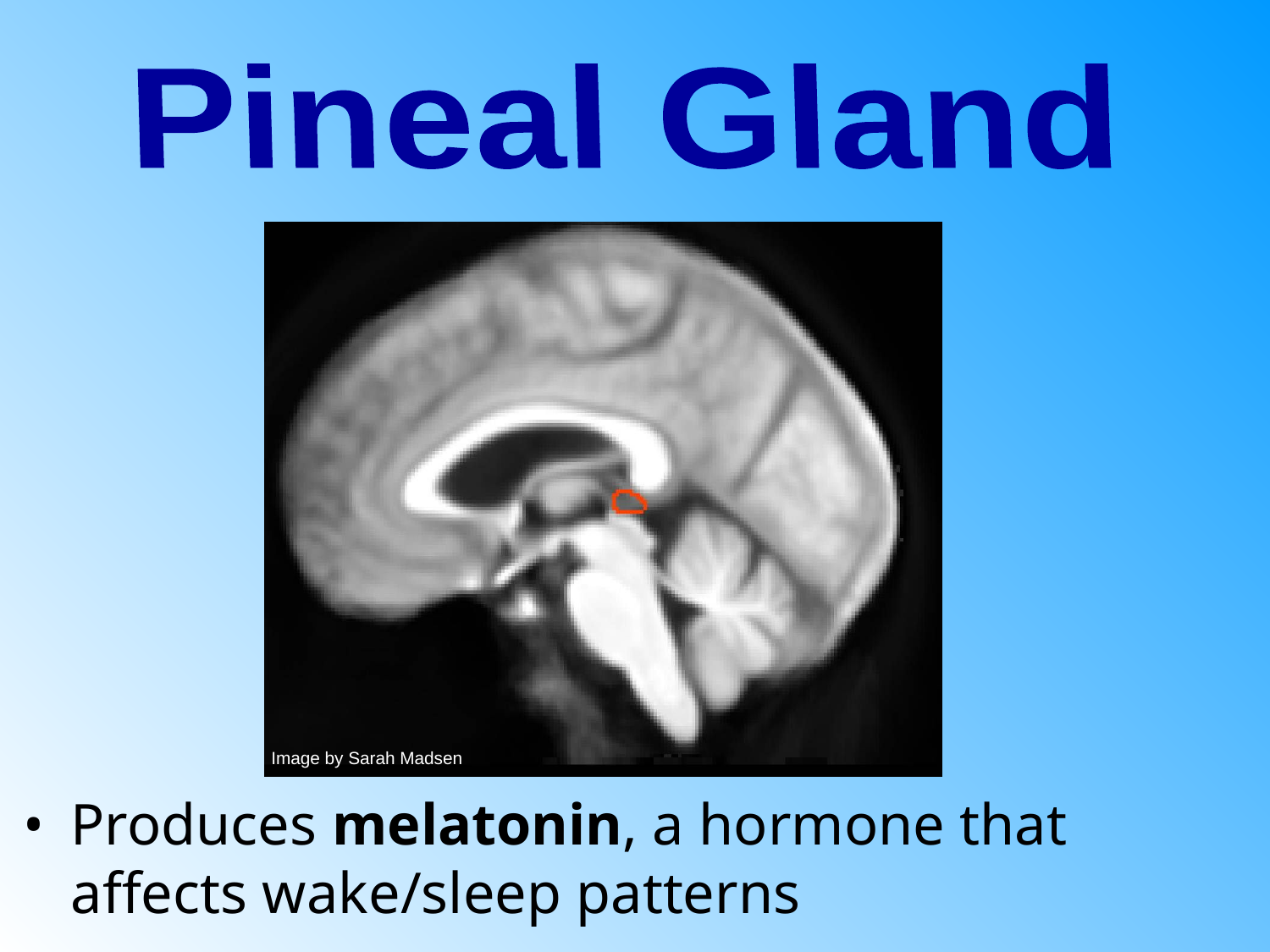

Pineal Gland
Image by Sarah Madsen
Produces melatonin, a hormone that affects wake/sleep patterns

## Slide 11
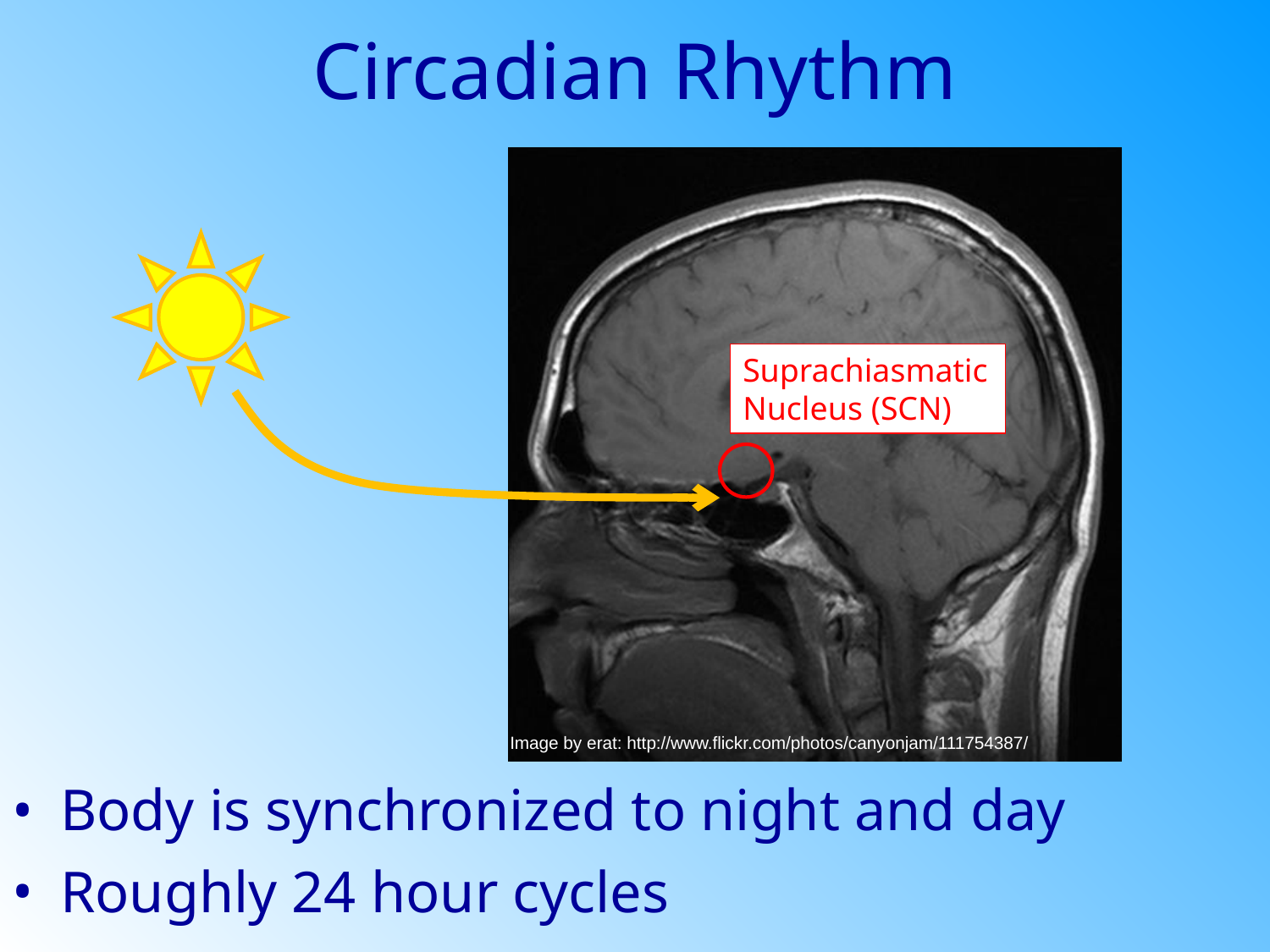

# Circadian Rhythm
Suprachiasmatic Nucleus (SCN)
Image by erat: http://www.flickr.com/photos/canyonjam/111754387/
Body is synchronized to night and day
Roughly 24 hour cycles

## Slide 12
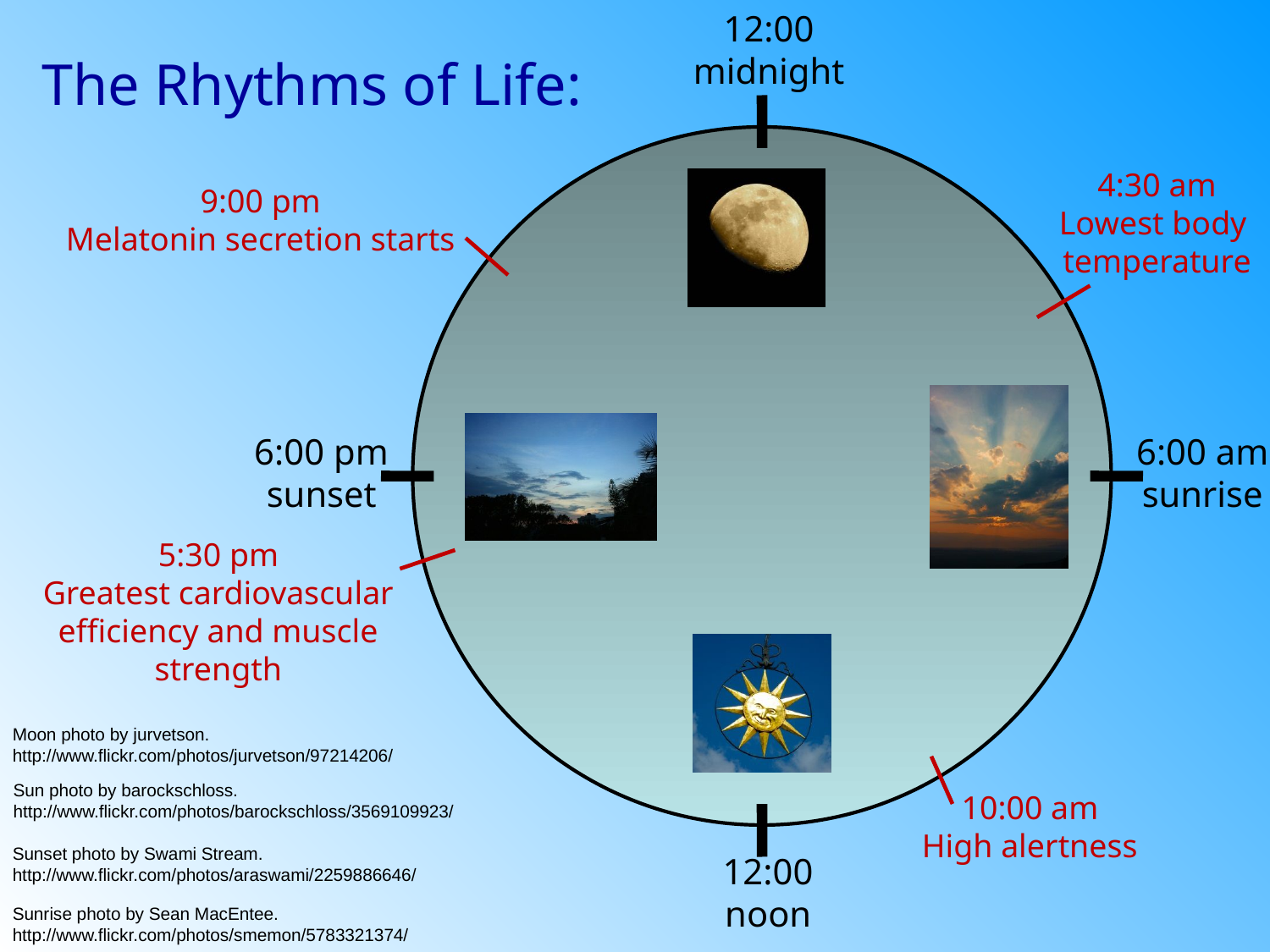

12:00
midnight
The Rhythms of Life:
4:30 am
Lowest body
temperature
9:00 pm
Melatonin secretion starts
6:00 pm
sunset
6:00 am
sunrise
5:30 pm
Greatest cardiovascular
efficiency and muscle strength
Moon photo by jurvetson.
http://www.flickr.com/photos/jurvetson/97214206/
Sun photo by barockschloss.
http://www.flickr.com/photos/barockschloss/3569109923/
10:00 am
High alertness
Sunset photo by Swami Stream. http://www.flickr.com/photos/araswami/2259886646/
12:00
noon
Sunrise photo by Sean MacEntee. http://www.flickr.com/photos/smemon/5783321374/

## Slide 13
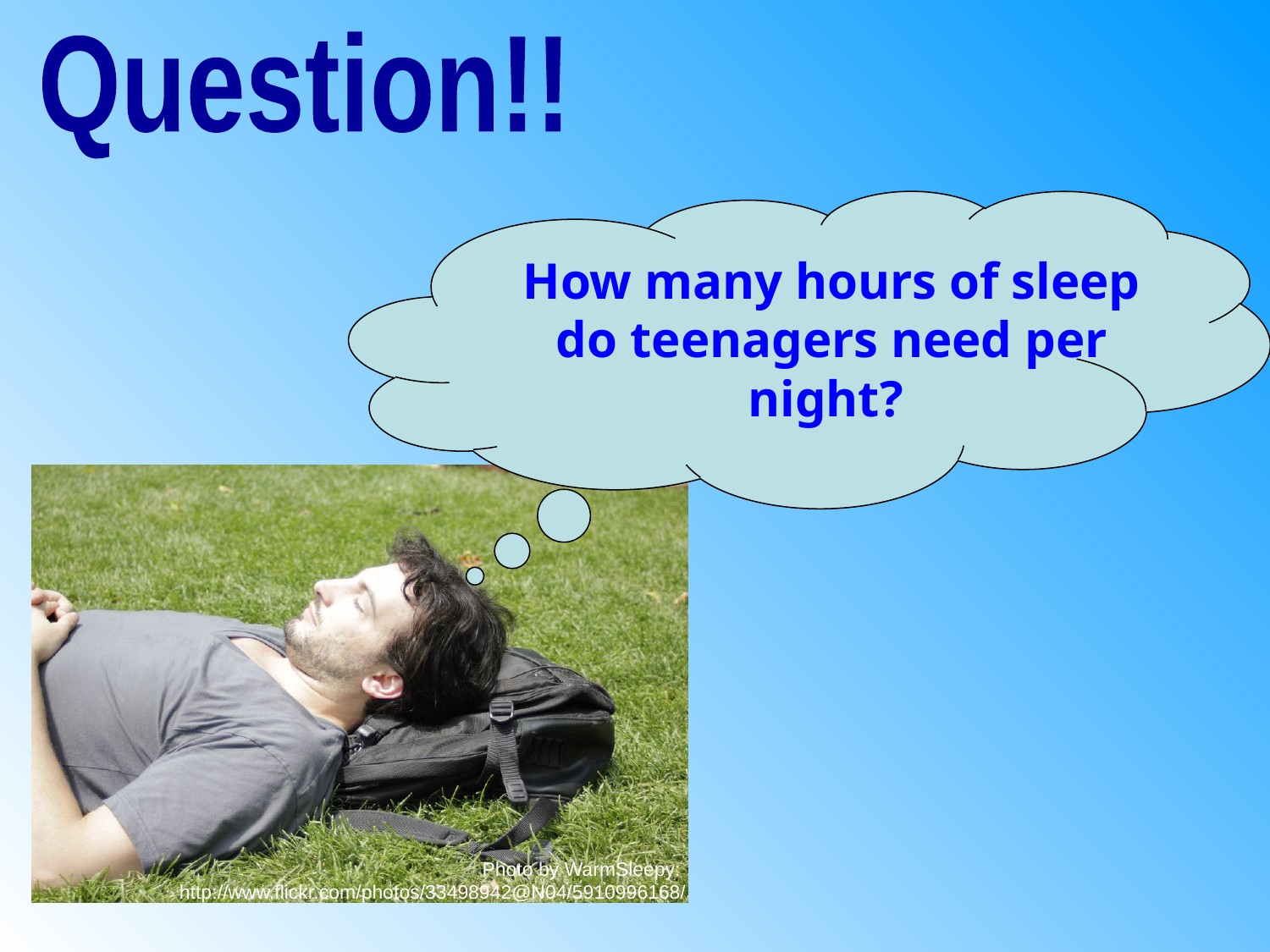

Question!!
# How many hours of sleep do teenagers need per night?
Photo by WarmSleepy:
http://www.flickr.com/photos/33498942@N04/5910996168/

## Slide 14
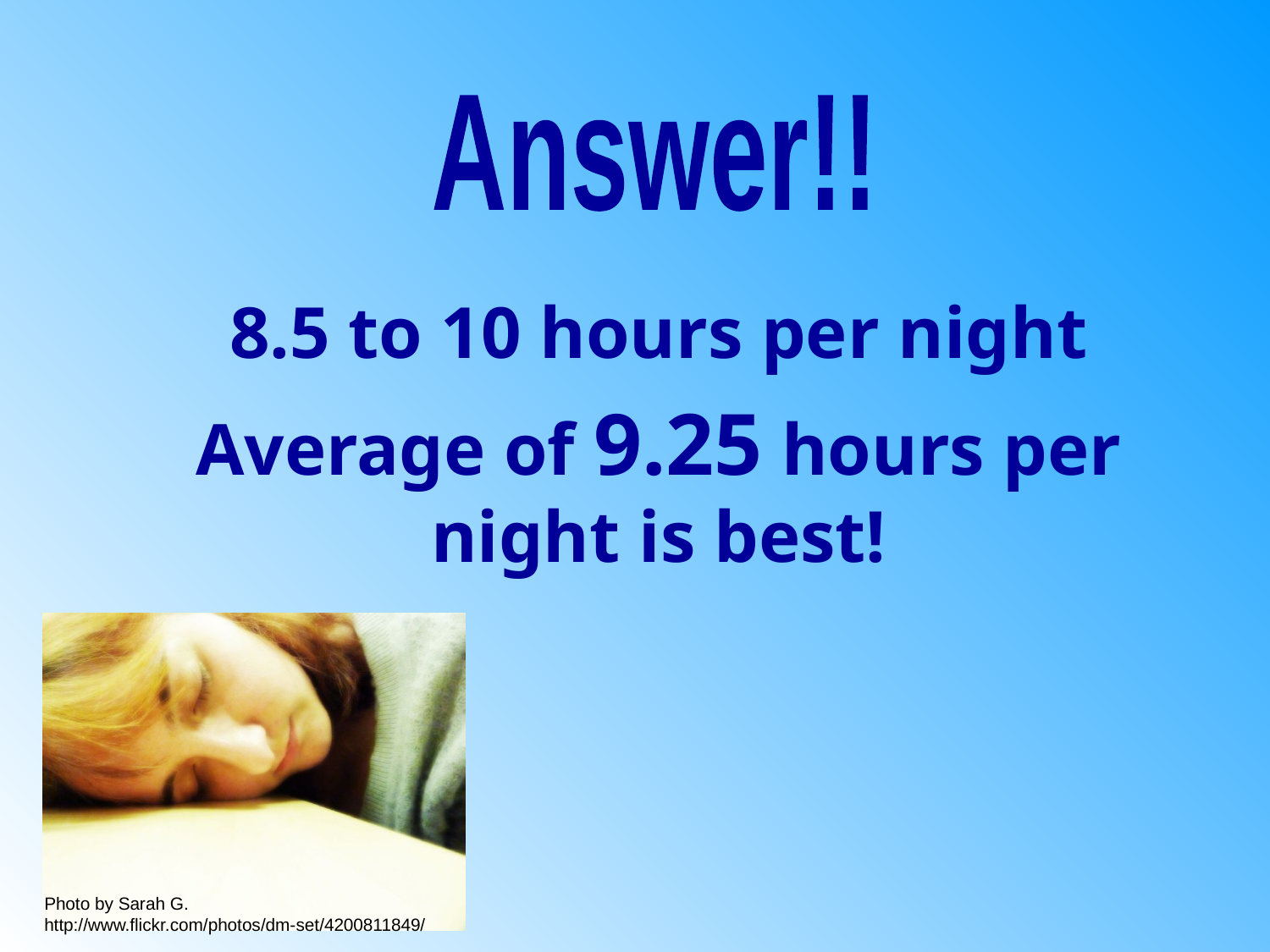

Answer!!
# 8.5 to 10 hours per night
Average of 9.25 hours per night is best!
Photo by Sarah G. http://www.flickr.com/photos/dm-set/4200811849/

## Slide 15
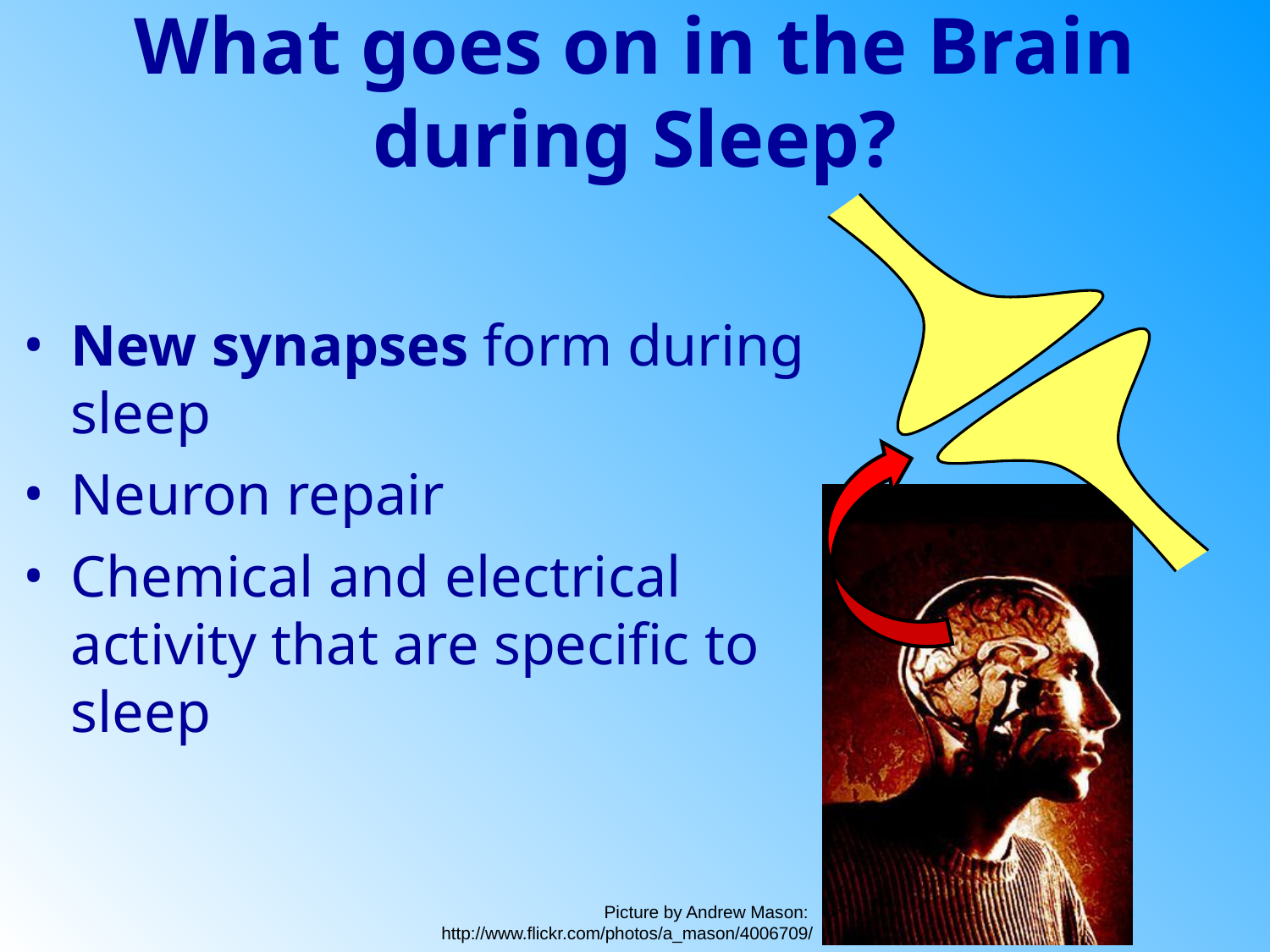

# What goes on in the Brain during Sleep?
New synapses form during sleep
Neuron repair
Chemical and electrical activity that are specific to sleep
Picture by Andrew Mason:
http://www.flickr.com/photos/a_mason/4006709/

## Slide 16
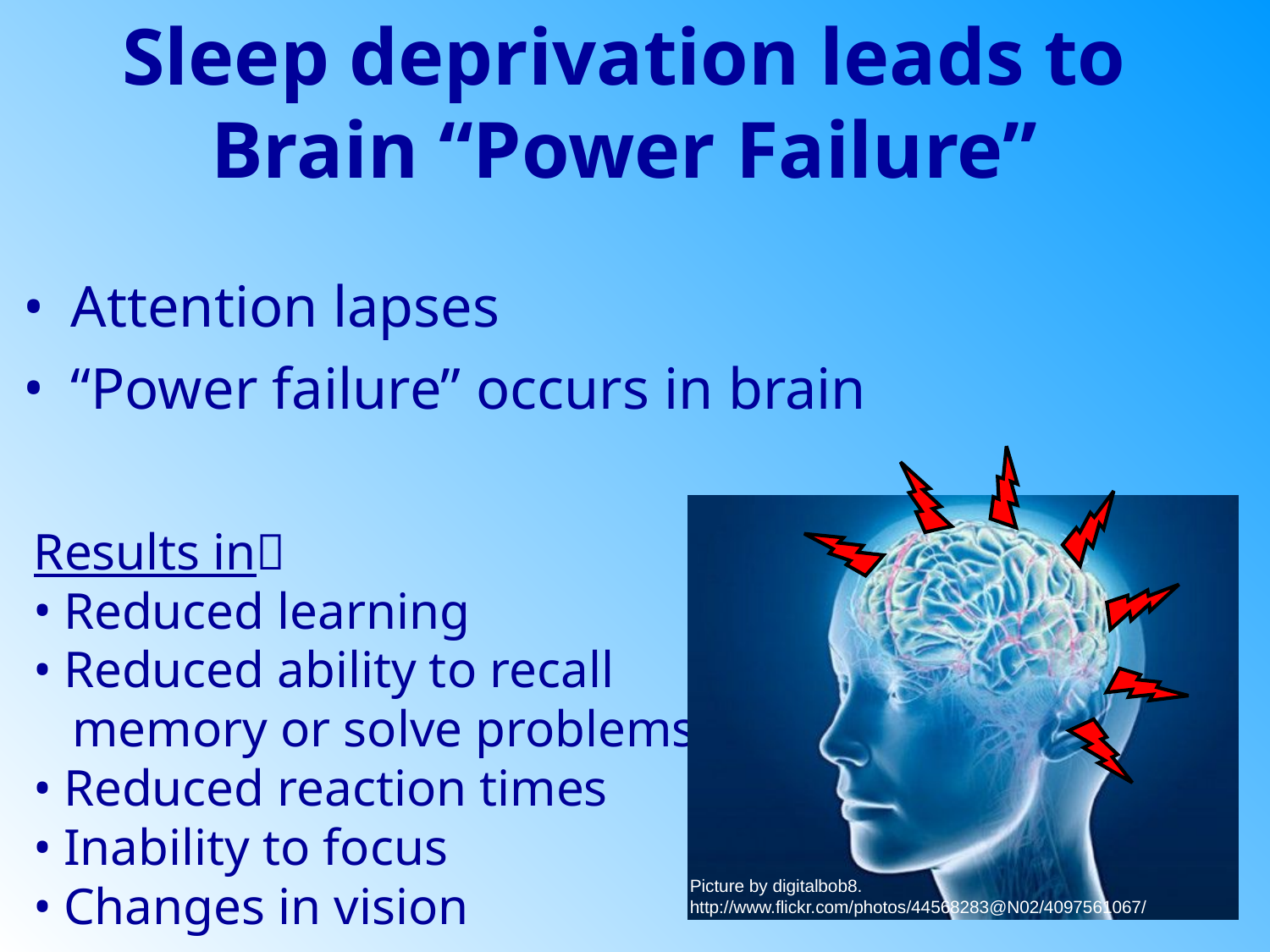

# Sleep deprivation leads to Brain “Power Failure”
Attention lapses
“Power failure” occurs in brain
Results in
Reduced learning
Reduced ability to recall
 memory or solve problems
Reduced reaction times
Inability to focus
Changes in vision
Picture by digitalbob8.
http://www.flickr.com/photos/44568283@N02/4097561067/

## Slide 17
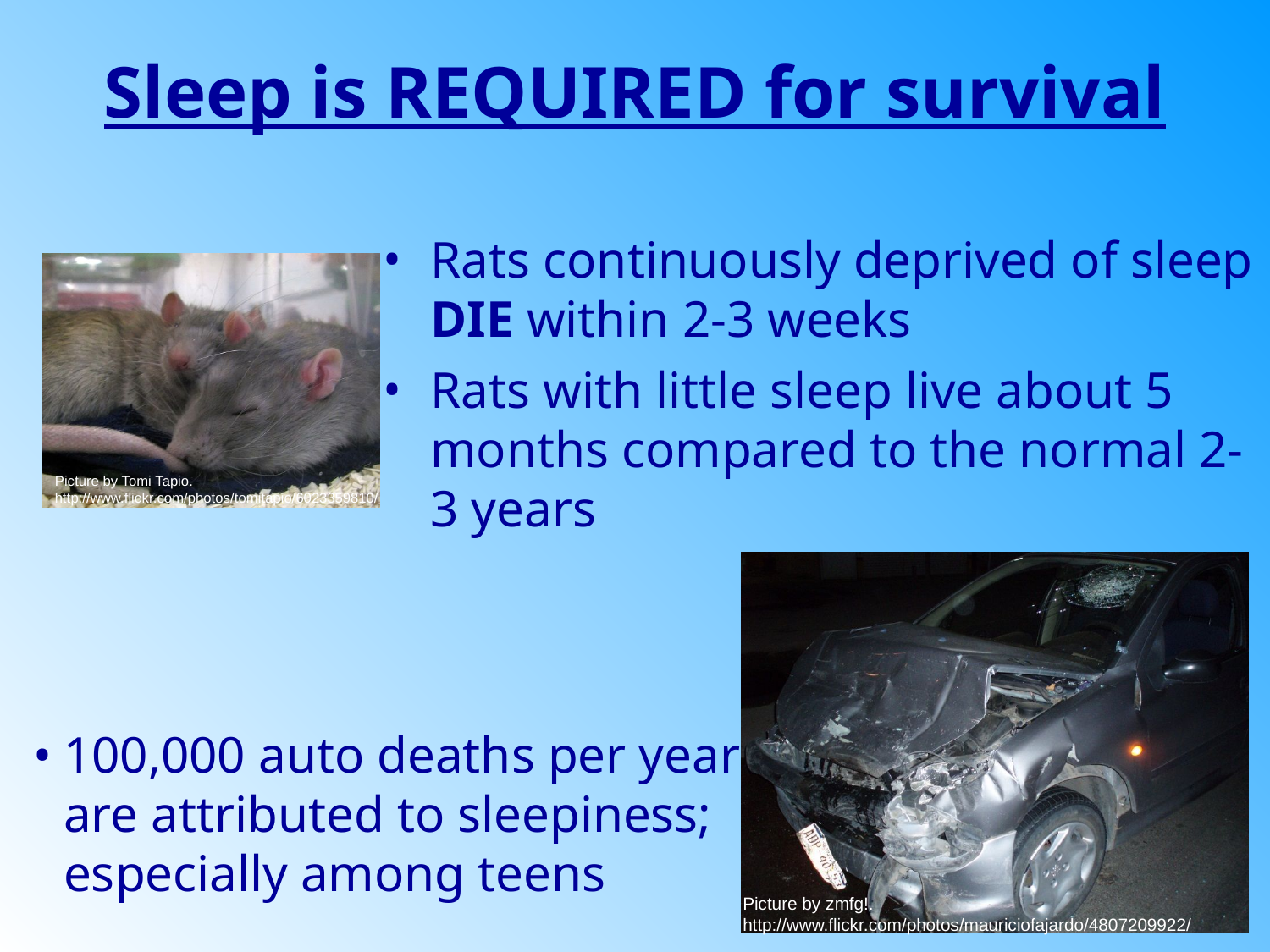

# Sleep is REQUIRED for survival
Rats continuously deprived of sleep DIE within 2-3 weeks
Rats with little sleep live about 5 months compared to the normal 2-3 years
Picture by Tomi Tapio.
http://www.flickr.com/photos/tomitapio/6023359810/
100,000 auto deaths per year are attributed to sleepiness; especially among teens
Picture by zmfg!.
http://www.flickr.com/photos/mauriciofajardo/4807209922/
