# Supplementary material for: Project Brainstorm: Using Neuroscience to Connect College Students with Local Schools
Source: PLoS Biol. 2012 Apr 17;10(4):e1001310. doi: 10.1371/journal.pbio.1001310 (PMC3328426; doi:10.1371/journal.pbio.1001310)
Supplement: Text S3 — Example brain-in-perspective topics for three different age groups. (RTF) [file pbio.1001310.s004.rtf]

Text S3.

Examples of Brain-in-Perspective Topics For Three Different Student Age Groups.


A) Topic for the 5-9 years of age student group:


Brain Injuries & Brain Protection
Background: 
The purpose of this activity is to illustrate the importance of wearing protective head gear while doing outside activities such as riding a bike or skateboard or while playing sports like football or hockey, and to show how some brain injuries, specifically concussions, occur and how they effect the brain. We will conclude by describing some of the body's built in brain protecting structures such as the skull, dura/pia matter, cerebral spinal fluid, and explain their role in preventing an injury.

Materials for Activity:  	
·	1 empty jar.
·	1 jar filled with water.
·	2 eggs.
·	4 colors of modeling clay (enough for 20 students).

Outline: 
1)	Begin by explaining what a concussion is in terms the audience can understand, basically a brain bruise, and how it can affect our senses and brain activity.
2)	Continue by showing how a car or sports accident can cause such an injury by explaining the three collisions of any accident or fall. The first being the car or helmet hitting something, the second being you hitting the car, ground, or helmet, and the last being your insides, including your brain, hitting your skull.
3)	Begin Activity: With the various colors of play-do representing the different structures, build a model of the head to demonstrate the order of the layers (brain  pia matter  dura matter  skull) and describe their function.
4)	While students build their own models, begin egg in a jar demo. By representing the egg as our brain and the jar as our skull, we can show the importance of a buffering fluid such as water, which will represent Cerebral Spinal Fluid.  Shake both the jar with the egg and the jar with the egg and water.  The jar with the egg only should break pretty easily while the egg in the jar with water should remain intact, thus demonstrating our point.
5)	Incorporate the results of the demo to the original topic of brain injury by asking related questions.
6)	Discuss the importance of both built in protections and things we can do to prevent serious head/brain injuries.
Take Home Message: 
Hopefully these interactive activities sufficiently explain what a concussion is, how the layers of the brain, including CSF, protect the brain from such injuries, and most importantly, engage the audience and help them understand the importance of seat belts and helmets. 


B) Topic for the 10-13 years of age student group:


Sleep Deprivation and Reaction Times

Background:
Research conducted on the effects of sleep deprivation on the brain has shown evidence that reaction times are reduced in individuals who are sleep deprived, versus individuals who are not. This result supports the fact that a full night's sleep is crucial for proper brain functioning. The purpose of this activity is to correlate reaction times with the hours of sleep the students have had. Two different test formats will be administered to expose the students to a variety of reaction time tests. Student performance on one may be different than the other, as some people may not be accustomed to computer usage, while another may be better at taking manual tests. 

Materials for Activity:
The manual reaction time test: 	
·	2-3 rulers, depending on the number of students. 
·	1 reaction time conversion table (see below).

The computerized reaction time test: 
·	a publicly available version of a reaction time test (example: http://faculty.washington.edu/chudler/java/redgreen.html).

Outline:
The manual reaction time test:
1)	Pair the students. One will hold the ruler, and the other will be the catcher (the catcher is the one taking the reaction time test).
2)	Have the catcher prepare to catch the ruler by positioning his or her thumb and index finger just under the end of the ruler.
3)	The person holding the ruler will hold the ruler vertically just above the catcher's thumb and index finger, and drop it without giving warning.
4)	The catcher will catch the ruler with thumb and index finger. The distance on the ruler at which the catcher catches the ruler can than be calculated into his or her reaction time by using a the conversion table below.
5)	If time permits, perform the test 5 times for each student, then average the reaction times.
6)	Ask the student the number of hours they've slept on average for the past few days and correlate that with their average reaction time. 


DISTANCE – REACTION TIME CONVERSION TABLE
Catch 						Catch
Distance      	Reaction time	   		Distance      Reaction time 
(cm)	        	(milliseconds)			(cm)	        (milliseconds)

1		50				16		180
2		60				17		190
3		70				18		190
4		80				19		200
5		90				20		200
6		100				21		210
7		120				22		210
8		130				23		220
9		140				24		220
10		140				25		230
11		150				26		230
12		160				27		230
13		160				28		240	
14		170				29		240	
15		170				30		250	

REACTION TIME RATING
 
Reaction time	Rating		Comment
(milliseconds)

0-50		Ultra-fast	A clairvoyant catcher… are you cheating?!
50-130		Superb		Impressive, do you play computer games? Next stop, F16!
131-175		Excellent	Well done, are you a text messager?
176-200		Good		Keep trying, you're not top gun yet!
201-240		Average		Not bad – but you're just Joe Average.
241-250		Fair		You'd get faster if it were money instead of a ruler!
251+		Slow		…ouch! Did the ruler hit your foot? Keep trying!


The computerized reaction time test:
1)	Download a publicly available reaction time test onto a laptop computer. 
2)	Have the students take the test. For the red-light, green-light test, the students click on a button when the light on the screen switches from red to green, and the computer automatically calculates the time it took for the student to click the button as the reaction time. 
3)	Have the student repeat the button clicking for a total of 5 trials (the computerized test automatically has 5 trials), and the computer will automatically average the reaction time test.
4)	Ask the student the number of hours they've slept on average for the past few days and correlate that with their average reaction time. 

Take Home Message:
The central idea is that sleep is essential for healthy and proper functioning of the brain. When the brain is sleep-deprived, it is often underperforming. It is essential that individuals get an equal amount of sleep for the optimal number of hours every night for the brain to perform at its best each day. 


C) Topic for the 14-18 years of age student group:


Nerve Impulse Conduction: The Importance of Myelin

Background: 
Myelin plays an important role in signal propagation, like in the sensation and perception of pain. It insulates axons and allows for more efficient message transmission, much in the same way that insulation on an electrical wire helps with the transmission of electricity. Pain pathways are predominately mediated by two types of nerve fibers – A delta fibers that transmit sharp fast pain, and C fibers that transmit slow dull pain. The characteristic that enables these differences in pain transduction is myelin. Myelinated A delta fibers are able to transmit information faster, causing the brain to perceive the pain as sharp and fast. In contrast, unmyelinated C fibers transmit information less promptly, a signal the brain interprets as slow dull pain.  

Materials for Activity: 
·	50 Straws
·	5 Safety pins
·	1 roll of electrical tape
·	20 Cups, large and small
·	2 Trays

Outline:
1)	Begin the activity by reintroducing myelin and its role in signal transduction.
2)	Explain the differences between A delta fibers and C fibers, emphasizing the type of pain each transmits.
3)	Split students into two teams: Team A delta and Team C. Team C receives straws in which holes have been punched (using a safety pin prior to the activity) into the bottom half of the straw. Team A receives similarly treated straws; however, these straws have been insulated with electrical tape to simulate myelin, making them less “leaky” to signal.
4)	Assign each student to a station with one large cup, one small cup, and a tray over which to conduct the activity. Fill the large cup with water and place on it the left side of the tray. Leave the small cup empty and place it on the right. 
5)	For the activity, students from Team A delta and Team C race to fill the small cup with water from the large cup using their team-specific straws. The “myelinated” A delta straws generally transmit water (i.e. signal) more efficiently, and Team A delta usually wins. However, valiant effort from Team C sometimes leads to “unmyelinated” victory. Use this to segue into a discussion about factors outside of myelin, such as input quantity and strength, that modulate transduction speed and efficiency.  

Take Home Lesson: 
Pain is mediated by different nerve fibers with different inherent properties, and signal conduction along each leads to different experiences of pain. Myelin, with its ability to insulate axons and thus transmit signal more efficiently, plays a particularly important role in these pain pathways, often mediating sharp sudden pain that requires immediate attention.
 
