# Supplementary material for: Project Brainstorm: Using Neuroscience to Connect College Students with Local Schools
Source: PLoS Biol. 2012 Apr 17;10(4):e1001310. doi: 10.1371/journal.pbio.1001310 (PMC3328426; doi:10.1371/journal.pbio.1001310)
Supplement: Text S4 — Example of a complete lesson plan for use in schools by teachers. (RTF) [file pbio.1001310.s005.rtf]

Text S4.

Example of a Complete Lesson Plan For Use in Schools by Teachers.

The following is an example of a lesson plan for elementary school children (5-9 years) that was prepared by 3rd and 4th year undergraduate students in the neuroscience department at UCLA. The brain-in-perspective topic is the 'structure and function' of the cerebral cortex and the lesson plan can be accompanied by standalone pictures, black/whiteboard drawings, props, a digital presentation or a combination of these. The lesson plan is divided into a 15 minute lecture presentation and a 30 minute hands-on practicum that will allow the students to practice identifying the location and function of the different lobes of the cerebral cortex.
______________________________________________

PRESENTATION (15 minutes)

Background:
Being young school students, we are safely assuming that our audience has had limited exposure to learning about the brain. Therefore our goal in this lesson plan is to get across how awesome and amazing our brain is by showing them all the things it is capable of doing! We start off with a very general introduction to the different parts of the brain and their respective functions, give a brief overview of the neuron, and end with a lesson on how the brain is like a muscle and needs to be kept healthy.

Outline:
1.	Begin with a question to lay a background of what they already know.
a.	What do you guys know about the brain? 
2.	On a long roll of paper, make a list of the tasks your brain is responsible for to exemplify how magnificent of an organ the brain is (to be prepared before teaching the lesson plan). Compile a real-time list by asking the kids some of the things they think they brain does. Write the list on the board. Participating is very key in this lesson.
3.	After they have had the chance to think a little about what the brain is capable of doing, unroll the previously prepared list. The roll of paper will already have a very long list of activities to make a clear point that it does all of what they said plus more!!
4.	Now that the complexity of the brain has been introduced, show how our brain needs to be organized in order to accomplish all these various tasks.
a.	Ask a volunteer to come up. Explain that you're going to throw a ball, and the student is going to catch it. Ask that as he/she catches it for the students to be paying attention to every detail and think about the small things he/she does to be able to catch the ball. 
b.	After the activity, ask the students what are some things you noticed that the student did?
c.	You will then rephrase it all and say: he/she has to first see the ball, move his/her hand to the right position, make a fist to grab the ball etc….All this takes a lot of planned out organization to accomplish it correctly. Also explain why it is important for the brain to do all these steps in ORDER.
5.	Explain that the brain is made up of a lot of very tiny cells that are called neurons. Ask them to repeat 'neurons'. Explain that the brain is made up of billions of neurons! Using a skittle as an analogy to a single neuron, ask them to imagine a handful of skittles, then a roomful of skittles, then the entire school full of skittles. This will get across to them that there are A LOT of neurons in the brain!
6.	Next, explain what neurons do. The neurons are the cells that work together to communicate to each other and relay messages across the brain. A neuron receives the information, processes it, and sends it along to the next neuron until it reaches the right part of the brain. 
7.	To exemplify this concept, ask a volunteer to come up. Ask that the student stretch out his/her arm. Using his/her arm and hand, point out if this was a neuron, which parts would be the dendrites, axons, and cell body and the function of the different parts. Ask that they repeat the words.
8.	Next, show them a picture of a real neuron, so that they do not associate neurons with just diagrams. Ask them to label the different parts. This will reinforce the fact that neurons are real and also enforce learning the different parts of the neuron.
9.	Explain that each side of the brain (technically the cerebral cortex) is divided into 4 parts (frontal, parietal, occipital and temporal lobes). Also mention the cerebellum and the brain stem. Together, this will cover the basic parts of the surface anatomy of the brain. Make sure that you explicitly tell the students that each of these parts has distinct functions.  
10.	Show a picture/poster of the cerebral lobes, cerebellum and brain stem, and repeat their main function. Ask them to point to that part of the brain and say the name of the part.
11.	Using the analogy that the brain is like a muscle, explain that the more you practice a task, the 'stronger' (better) you brain gets at that task!
12.	Make a statement that they can be good at whatever subject they want, they just need to work hard.
13.	Discuss how damaging different parts of the brain causes different problems (ex. damage to the temporal lobe might cause memory loss).
14.	To end on a more positive note, tell the students that a well protected brain is a happy brain.
15.	Using the analogy that the brain is like a muscle, explain that the more you practice a task, the 'stronger' (better) you brain gets at that task!
16.	Wrap up by asking what they have learned today.

Take home lessons of presentation:
1.	The brain is composed of several different parts. Each of these parts need to work together in order to let us accomplish the numerous different tasks that we perform every day.
2.	The different parts of the brain work together with the help of cells called neurons. The neuron is made up of dendrites, a cell body, and the axon. Its main function is to help different parts of the brain to communicate with each other.
3.	The brain (cerebrum) is made up of four parts called lobes: the temporal lobe, the occipital lobe, and parietal lobe, and the frontal lobe (+ a cerebellum and a brain stem). The temporal lobes are responsible for letting us hear sounds and to help us remember things; the occipital lobes are responsible for letting us see things; the parietal lobes control our senses and lets us process numbers and space; and the frontal lobes control our movements and also let us plan, solve problems, and make decisions.
4.	The brain is flexible like a muscle and can be kept strong and healthy with exercise. This means that your brain can accomplish almost anything you want; you just have to practice hard. 


PRACTICUM (30 minutes)

Background:
The goal of this practicum is to further show kids how different parts of the brain are responsible for different tasks. Using a bowl as the “brain cap”, the students will label the different parts of the brain and decorate with images of what their brain is doing in that lobe!

Materials:
1.	Sheets of paper cut up into 2x3 in rectangles
2.	Styrofoam bowls that they can flip over to use as brain caps
3.	Pieces of twine, cut into 2 or 3 in. strips
4.	Pens, markers, crayons

Outline:
1.	Have the kids draw a rough representation of the brain on their Styrofoam bowls by drawing a line down the middle and also the squiggly lines on the sides. 
2.	Ask each kid what he/she likes to do or is good at and write the activity a sheet of paper. If there is time, the child can also draw the activity underneath the word.
3.	Ask the kid which part of the brain they think is responsible for that activity. Refer back to the big brain drawing on the wall of the classroom that the lesson was given on.
4.	Help the kid insert the sheet of paper into the appropriate section of the head on the Styrofoam bowl using the strips of twine.
5.	Repeat until the end of the allotted time. By the end, each child should have at least 3 or 4 “flags” on their bowls. 
6.	Ask them to turn the bowls around and put it on their head to turn them into “braincaps”! 


Take home lessons:
The practicum will hopefully allow them to understand that the brain is a powerful organ that allows them to accomplish all the numerous tasks they perform throughout the day. Ideally, they will all have different tasks that they are good at and enjoy, and can compare with each other their different strengths. This will show them that they all have different brain structures. 
